# Supplementary material for: Ring-Modified Histidine-Containing Cationic Short Peptides Exhibit Anticryptococcal Activity by Cellular Disruption
Source: Molecules. 2022 Dec 22;28(1):87. doi: 10.3390/molecules28010087 (PMC9821961; doi:10.3390/molecules28010087)
Supplement: Supplementary file 1 [file molecules-28-00087-s001.zip › molecules-2053903-supplementary.pdf]

1. General Experimental

All the reagents and starting materials were procured from commercial sources such as Sigma-Aldrich, Alfa Aesar, Merck, TCI, Avra synthesis, Himedia, Novabiochem and Chem-Impex International and were without any purification unless otherwise stated. AR, LR and HPLC grade solvents were obtained from Rankem, JT Bakers and Merck. Analytical grade solvents were utilized for reactions whereas HPLC grade solvents for preparative and analytical HPLC. Solvents were dried by using standard procedures and stored over activated molecular sieves with positive nitrogen pressure and were used without any further purifications. Thin layer chromatography (TLC) was performed on Merck pre-coated silica gel plates (0.25 mm, 60 Å pore size) impregnated with a fluorescent indicator (254 nm) and 0.2 mm Merck pre-coated alumina 60 F_{254} Aluminum sheets. Visualization on TLC was done either under UV light (254 nm), or staining with iodine or by treatment with 10% ninhydrin in ethanol or by ethanolic solution of phosphomolybdic acid (5% v/v, a universal indicator) followed by heating. Reactions under microwave irradiation were done on CEM Discover® microwave reactor. Synthesized compounds were purified by fully automated Biotage® purification system flash chromatography on Aldrich silica gel (230–400 mesh) or neutral alumina (Brockman I) or by using column chromatography with silica gel (60–120 mesh or 100–200 mesh) or neutral alumina (Brockman I). Shimadzu Prominence preparative HPLC system was utilized for isolation of certain peptides using a Phenomenex C-18 column (250 × 21 mm, particle size = 10 µm). All final peptides were checked for their homogeneity and purity on the Shimadzu Prominence A-8 HPLC system using a Phenomenex Gemini® LC-18 column (25 cm × 4.6 mm, particle size = 5 µm, pore size = 110 Å). The peptides were analyzed by using solvent system of CH_3CN-H_2O-0.1% trifluoroacetic acid (TFA) in gradient flow of 5–95% at 210 or 254 or 280 nm for 40 min. High-resolution mass spectra (HRMS) were recorded on Agilent 6546 LC/Q-TOF in the ESI mode. Nuclear magnetic resonance spectra (NMR) were recorded on a Bruker Avance-III 400 spectrometer (^{1}H NMR, 400 MHz and ^{13}C NMR, 100 MHz) or Jeol ECA-500 spectrometer (^{1}H NMR, 500 MHz and ^{13}C NMR, 125 MHz). Chemical shifts of the data for ^{1}H-NMR were reported as δ values in ppm and coupling constants (*J*) were in hertz (Hz) relative to tetramethylsilane (TMS). The ^{1}H-NMR data is reported as follows: chemical shift, spin multiplicity, coupling constant and integration. The following abbreviations were used for spin multiplicity: br = broad, s = singlet, d = doublet, t = triplet, dd = double doublet, m = multiplet, q = quadruplet and if splitting patterns could not be interpreted easily are reported as multiplet (m). Chemical shifts for ^{13}C-NMR were reported in ppm relative to the solvent peak.

2. Synthesis

2.1. Synthesis of N-α-Boc-L-Histidine Methyl Ester (4)

L-His-OMe (**3**) was weighed under air in a pre-dried round bottom flask and methanol (50 mL) was added to it, followed by addition of triethylamine (2 equiv.) and Boc anhydride (2.3 equiv.). The reaction mixture was stirred for 2 h at ambient temperature. The solvent was completely removed under reduced pressure and water (30 mL) was added to the crude mixture followed by extraction with dichloromethane. The organic layer was separated and solvent was removed under reduced pressure. The crude product N- α-(*tert*-butoxycarbonyl)-1-*tert*-butoxycarbonyl histidine methyl ester was dissolved in methanol (30 mL) followed by addition of K_2CO_3 (0.1 equiv.) and the reaction mixture was refluxed for 3 h. The reaction mixture was dried under reduced pressure and purified by column chromatography using CH_2Cl_2 (90- 92%) and CH_3OH (8–10%). The desired product Boc-His-OMe (**4**) was obtained in 85% yield.[1]

***N*-α-Boc-L-histidine methyl ester (4):** Yield: 85%; ^{1}H-NMR (400 MHz, $CDCl_3$): δ 7.54 (s, 1H), 6.81 (s, 1H), 5.84 (d, *J* = 7.6 Hz, 1H), 4.54 (d, *J* = 6.8 Hz, 1H), 3.69 (s, 3H), 3.09 (d, *J* =

4.4 Hz, 2H), 1.43 (s, 9H); ^{13}C-NMR (100 MHz, $CDCl_3$): δ 172.7, 155.7, 135.3, 133.9, 116.2, 79.9, 53.6, 52.3, 29.7, 28.3; ESI-TOF: *m/z* $(M + H)^+$ $C_{12}H_{19}N_3O_4$: 270.30.

2.2. Synthesis of Boc-His(1-Bzl, 5-iodo)-OMe (7a-o, Scheme 1, Figure 2)

The reaction was carried out in 2 steps: *C*-5 iodination followed by *N*-1 benzylation reaction. Boc-His- OMe (1 equiv.) was used as starting material and it was weighed under air in a two necked round bottom flask with magnetic stirring bar. The starting material was dried under vacuum and was dissolved in CH_3CN (50 mL) under positive argon pressure. *N*-Iodosuccinimide (1.2 equiv.) was added portion wise under dark and inert conditions. The reaction was stirred for 24 h at ambient temperature. The reactionwas monitored by TLC and after completion, saturated solution of sodium thiosulfate was added followed by further stirring for 10–15 min in order to quench excess of iodine. The solvent from the reaction mixture was dried under reduced pressure and water was added. The crude product was extracted with ethyl acetate and organic layer was concentrated under reduced pressure. Purification was done using silica gel (100–200) column chromatography and pure product **6** was isolated in 70% yield.[4]

Further, *N*-1 benzylation of **6** was carried out by reaction with substituted benzyl bromides. Boc-His(1- Bzl)-OMe (1 equiv.) was weighed under air in a pre-dried round bottom flask (50 mL) equipped with magnetic stirring bar and dried under vacuum followed by back filling with nitrogen. The starting material was dissolved in dried DMF (5 mL) followed by the addition of K_2CO_3 (1.5 equiv.) underpositive nitrogen pressure and the reaction was stirred for 30 min at ambient temperature. Substituted benzyl bromide (1.5 equiv.) was added to the mixture after 30 min and the reaction was further stirred for 4–6 h at ambient temperature. After completion of the reaction, the solvent was removed under reduced pressure and water was added followed by extraction with ethyl acetate. The organic layer was separated and dried under reduced pressure followed by purification using automated flash chromatography system (Biotage) by gradient run of ethyl acetate (10–15%) and hexane (85–90%).[3] The final derivatives (**7a-o**) were isolated in 65–90% yield.

***N*-α-Boc-5-iodo-L-histidine methyl ester (6):** Yield: 75%; ^{1}H-NMR (400 MHz, $CDCl_3$): δ 7.64 (s, 1H), 5.84 (d, *J* = 7.6 Hz, 1H), 4.54 (d, *J* = 6.8 Hz, 1H), 3.69 (s, 3H), 3.09 (d, *J* = 4.4 Hz, 2H), 1.43 (s, 9H); ^{13}CNMR (100 MHz, $CDCl_3$): δ 172.1, 155.6, 139.3, 114.1, 80.7, 52.9, 50.7, 33.8, 32.0, 31.9, 29.7, 29.5, 29.4, 29.2, 28.9, 28.3, 22.7, 14.1; MS (ESI-TOF): *m/z* $(M + H)^+$ 396.05.

***N*-α-Boc-1-benzyl-5-iodo-L-histidine methyl ester (7a):** ^{1}H-NMR (400 MHz, $CDCl_3$): δ 7.67 (s, 1H), 7.46–7.32 (m, 3H), 7.08 (d, *J* = 4 Hz, 2H), 6.06 (d, *J* = 8.4 Hz, 1H), 5.09 (s, 2H), 4.64–4.59 (m, 1H), 3.69 (s, 3H), 3.16–3.11 (m, 1H), 3.02 (dd, *J* = 4.8, 10.0 Hz, 1H), 1.45 (s, 9H); ^{13}C-NMR (100 MHz, $CDCl_3$): δ 172.3, 171.5, 155.5, 142.7, 140.1, 135.4, 129.1, 128.9, 128.5, 128.2, 127.2, 127.1, 79.6, 72.0, 53.1, 52.3, 51.7, 49.7, 30.5, 28.4, 28.3; HRMS (ESI-TOF) m/z $(M + H)^+$ calculated for $C_{19}H_{24}IN_3O_4$ 486.0884, found 486.0906.

***N*-α-Boc-1-(4-*tert*-butylbenzyl)-5-iodo-L-histidine methyl ester (7b):** Yield: 78%; ^{1}H-NMR (400 MHz,$CDCl_3$): δ 7.65 (s, 1H), 7.38 (d, *J* = 8.0 Hz, 2H), 7.04 (d, *J* = 7.6 Hz, 2H), 6.06 (d, *J* = 8.4 Hz, 1H), 5.05 (s, 2H), 4.63–4.59 (m, 1H), 3.70 (s, 3H), 3.14 (dd, *J* = 5.2, 9.6 Hz, 1H), 3.03 (dd, *J* = 4.8, 10.0 Hz, 1H), 1.45 (s, 9H), 1.32 (s, 9H); ^{13}C-NMR (100 MHz, $CDCl_3$): δ 172.3, 155.5, 151.3, 142.5, 140.0, 132.4, 126.9,125.9, 53.1, 52.3, 51.4, 34.6, 31.3, 30.5, 28.4; HRMS (ESI-TOF): *m/z* $(M + H)^+$ calculated for $C_{23}H_{33}IN_3O_4$ 542.1510, found 542.1530.

***N*-α-Boc-1-(4-*iso*-propylbenzyl)-5-iodo-L-histidine methyl ester (7c):** Yield: 80%; ^{1}H-NMR (400 MHz, $CDCl_3$): δ 7.65 (s, 1H), 7.22 (d, *J* = 8.4 Hz, 2H), 7.03 (d, *J* = 7.6 Hz, 2H), 6.05 (d, *J* = 8.8 Hz, 1H), 5.05 (s, 2H), 4.63–4.58 (m, 1H), 3.70 (s, 3H), 3.14 (dd, *J* = 5.2, 9.6 Hz, 1H), 3.02 (dd, *J* = 4.8, 10.0 Hz, 1H), 2.91 (sept, *J* = 6.8 Hz, 1H), 1.45 (s, 9H), 1.32 (d, *J* = 86.8 Hz, 6H); ^{13}C-NMR (100 MHz, $CDCl_3$): δ 172.3, 155.5, 149.1, 142.5, 140.0, 132.7, 127.2, 127.0, 79.6, 53.1, 52.3, 51.5, 33.8, 30.5, 28.4, 23.9; HRMS (ESI-TOF): *m/z* $(M + H)^+$ calculated for $C_{22}H_{31}IN_3O_4$ 528.1354, found 528.1375.

***N*-α-Boc-1-(3,5-di-*tert*-butylbenzyl)-5-iodo-L-histidine methyl ester (7d):** Yield: 82%; ^{1}H-NMR (400MHz, $CDCl_3$): δ 7.62 (s, 1H), 7.39–7.38 (m, 1H), 7.01 (d, *J* = 1.6 Hz, 2H),

6.03 (d, *J* = 8.4 Hz, 1H), 5.04 (s, 2H), 4.63–4.58 (m, 1H), 3.72 (s, 3H), 3.16 (dd, *J* = 5.2, 10.0 Hz, 1H), 3.04 (dd, *J* = 4.8, 10.0 Hz, 1H), 1.45 (s, 9H), 1.31 (s, 18H); ^{13}C-NMR (100 MHz, $CDCl_3$): δ 172.3, 155.6, 151.6, 142.3, 139.9, 134.2, 122.9, 121.9, 79.6, 72.0, 53.0, 52.4, 52.3, 34.9, 31.4, 30.4, 28.4, 27.3; HRMS (ESI-TOF): *m/z* (M + H)$^+$ calculated for $C_{27}H_{41}IN_3O_4$ 598.2136, found 598.2162.

***N*-α-Boc-1-(3-trifluoromethylbenzyl)-5-iodo-L-histidine methyl ester (7e):** Yield: 85%; ^{1}H-NMR (400 MHz, $CDCl_3$): δ 7.75 (br.s, 1H), 7.60 (d, *J* = 7.6 Hz, 1H), 7.52–7.48 (m, 1H), 7.41 (s, 1H), 7.19 (d, *J* = 6.8 Hz, 1H), 6.02 (d, *J* = 8.0 Hz, 1H), 5.18 (s, 2H), 4.64–4.62 (m, 1H), 3.71 (s, 3H), 3.17–3.12 (m, 1H), 3.06–3.02 (m, 1H), 1.45 (s, 9H); ^{13}C-NMR (100 MHz, $CDCl_3$): δ 172.3, 155.5, 143.2, 140.2, 136.5, 131.8, 131.6, 131.2, 130.9, 130.3, 129.7, 125.2, 125.1, 123.9, 123.8, 122.4, 119.7, 79.7, 72.0, 53.0, 52.3, 51.2, 31.9, 30.6, 29.7, 28.4; HRMS (ESI-TOF): *m/z* (M + H)$^+$ calculated for $C_{20}H_{24}F_3IN_3O_4$ 554.0758, found 554.0762.

***N*-α-Boc-1-(2-trifluoromethylbenzyl)-5-iodo-L-histidine methyl ester (7f):** Yield: 88%; ^{1}H-NMR (400 MHz, $CDCl_3$): δ 7.73 (d, *J* = 8.0 Hz, 1H), 7.51–7.41 (m, 2H), 6.52 (d, *J* = 7.6 Hz, 1H), 6.07 (d, *J* = 8.4 Hz, 1H), 5.33 (s, 2H), 4.66–4.62 (m, 1H), 3.71 (s, 3H), 3.18–3.14 (m, 1H), 3.08–3.03 (m, 1H), 1.46 (s, 9H); ^{13}C-NMR (100 MHz, $CDCl_3$): δ 172.3, 155.5, 140.2, 136.5, 131.6, 130.3, 129.6, 125.1, 123.8, 122.3, 79.7, 72.4, 53.1, 52.4, 51.2, 30.6, 29.5, 28.4; HRMS (ESI-TOF): *m/z* (M + H)$^+$ calculated for $C_{20}H_{24}F_3IN_3O_4$ 554.0758, found 554.0760.

***N*-α-Boc-1-(4-trifluoromethylbenzyl)-5-iodo-L-histidine methyl ester (7g):** Yield: 87%; ^{1}H-NMR (400 MHz, $CDCl_3$): δ 7.77 (s, 1H), 7.63 (d, *J* = 8.8 Hz, 2H), 7.17 (d, *J* = 8.4 Hz, 2H), 6.00 (d, *J* = 9.2 Hz, 1H), 5.19 (s, 2H), 4.64–4.59 (m, 1H), 3.71 (s, 3H), 3.15–3.02 (m, 2H), 1.45 (s, 9H); ^{13}C-NMR (100 MHz, $CDCl_3$): δ 172.3, 155.5, 140.2, 139.5, 130.7, 130.3, 127.1, 126.0, 125.9, 79.7, 53.0, 52.4, 51.1, 30.6, 29.7, 28.4; HRMS (ESI-TOF): *m/z* (M + H)$^+$ calculated for $C_{20}H_{24}F_3IN_3O_4$ 554.0758, found 554.0784.

***N*-α-Boc-1-(4-nitrobenzyl)-5-iodo-L-histidine methyl ester (7h):** Yield: 89%; ^{1}H-NMR (400 MHz,$CDCl_3$): δ 8.13 (d, *J* = 8.8 Hz, 2H), 7.41 (s, 1H), 7.16 (d, *J* = 8.8 Hz, 2H), 5.77 (d, *J* = 8 Hz, 1H), 5.10 (s, 2H), 4.50–4.45 (m, 1H), 3.59 (s, 3H), 3.02–2.96 (m, 2H), 1.34 (s, 9H); ^{13}C-NMR (100 MHz, CD_3OD): δ 172.5, 155.5, 147.8, 143.4, 138.9, 137.4, 135.2, 127.6, 125.0, 124.2, 116.9, 79.7, 53.5, 52.2, 49.9, 32.2, 30.4, 29.7, 28.3, 26.4, 23.4; HRMS (ESI-TOF): *m/z* (M + H)$^+$ calculated for $C_{19}H_{24}IN_4O_6$ 531.0735, found 531.0732.

***N*-α-Boc-1-(3,4-difluorobenzyl)-5-iodo-L-histidine methyl ester (7i):** Yield: 90%; ^{1}H-NMR (400 MHz, $CDCl_3$): δ 7.72 (s, 1H), 7.19–7.13 (m, 1H), 6.91–6.97 (m, 1H), 6.81–6.79 (m, 1H), 5.99 (d, *J* = 7.6 Hz, 1H), 5.07 (s, 2H), 4.64–4.59 (m, 1H), 3.71 (s, 3H), 3.12 (d, *J* = 5.6 Hz, 1H), 3.05–3.00 (m, 1H), 1.45 (s, 9H); ^{13}C-NMR (100 MHz, $CDCl_3$): δ 172.3, 155.5, 151.7, 151.4, 149.3, 140.0, 132.5, 123.0, 118.0, 116.0, 79.7, 53.0, 52.3, 50.6, 30.6, 29.7, 28.4; HRMS (ESI-TOF): *m/z* (M + H)$^+$ calculated for $C_{19}H_{23}F_2IN_3O_4$ 522.0696, found 522.0697.

***N*-α-Boc-1-(3,5-difluorobenzyl)-5-iodo-L-histidine methyl ester (7j):** Yield: 92%; ^{1}H-NMR (400 MHz, $CDCl_3$): δ 7.73 (s, 1H), 6.79–6.74 (m, 1H), 6.56 (d, *J* = 5.6 Hz, 2H), 6.00 (d, *J* = 2.1 Hz, 1H), 5.09 (s, 2H), 4.64–4.59 (m, 1H), 3.71 (s, 3H), 3.13 (dd, *J* = 5.6, 9.2 Hz, 1H), 3.03 (dd, *J* = 4.8, 9.6 Hz, 1H), 1.44 (s, 9H); ^{13}C-NMR (100 MHz, $CDCl_3$): δ 172.3, 164.7, 162.2, 155.5, 143.3, 140.1, 139.6, 109.9, 103.7, 79.7, 71.9, 53.0, 52.3, 50.6, 30.6, 29.7, 28.3; HRMS (ESI-TOF): *m/z* (M + Na)$^+$ calculated for $C_{19}H_{22}F_2IN_3NaO_4$ 544.0521, found 544.0519.

***N*-α-Boc-1-(3-fluorobenzyl)-5-iodo-L-histidine methyl ester (7k):** Yield: 75%; ^{1}H-NMR (400 MHz,$CDCl_3$): δ 7.78 (br.s, 1H), 7.37–7.31 (m, 1H), 7.05–7.01 (m, 1H), 6.86 (d, *J* = 6.8 Hz, 1H), 6.77 (d, *J* = 8.4 Hz, 1H), 6.03 (d, *J* = 6.4 Hz, 1H) 5.12 (s, 2H), 4.62 (br.s, 1H), 3.71 (s, 3H), 3.14 (d, *J* = 15.2 Hz, 1H), 3.04 (d, *J* = 11.6 Hz, 1H), 1.45 (s, 9H); ^{13}C-NMR (100 MHz, $CDCl_3$): δ 172.3, 164.3, 161.8, 155.5, 140.2, 137.9, 137.9, 130.7, 130.6, 122.6, 115.4, 115.2, 114.2, 113.9, 79.7, 53.0, 52.4, 51.3, 30.6, 29.7, 28.4; HRMS (ESI-TOF): *m/z* (M + H)$^+$ calculated for $C_{19}H_{23}FIN_3O_4$ 504.0790, found 504.0788.

***N*-α-Boc-1-(3-chlorobenzyl)-5-iodo-L-histidine methyl ester (7l):** Yield: 72%; ^{1}H-NMR (400 MHz, $CDCl_3$): δ 7.75 (br.s, 1H), 7.29 (d, *J* = 10.8 Hz, 2H), 7.09 (s, 1H), 6.94 (s, 1H), 6.04 (d, *J* = 7.2 Hz, 1H), 5.09 (s, 2H), 4.62 (br.s, 1H), 3.71 (s, 3H), 3.14 (d, *J* = 12.4 Hz, 1H), 3.03 (d, *J* = 11.6 Hz, 1H), 1.45 (s, 9H); ^{13}C-NMR (100 MHz, $CDCl_3$): δ 172.3, 155.5, 142.9, 140.2, 137.5, 134.9, 130.3, 128.5, 127.4, 125.1,79.7, 53.03, 52.4, 51.2, 30.6, 29.7, 28.4;

HRMS (ESI-TOF): *m/z* (M + Na)$^+$ calculated for $C_{19}H_{23}ClIN_3NaO_4$ 542.0319, found 542.0319.

***N-α*-Boc-1-(3-bromobenzyl)-5-iodo-L-histidine methyl ester (7m):** Yield: 74%; ^{1}H-NMR (400 MHz, $CDCl_3$): δ 7.74 (br.s, 1H), 7.46 (d, *J* = 7.6 Hz, 1H), 7.25–7.22 (m, 2H), 6.97 (d, *J* = 6.16 Hz, 1H), 6.04 (d, *J* = 7.6 Hz, 1H) 5.08 (s, 2H), 4.62 (br.s, 1H), 3.71 (s, 3H), 3.14 (d, *J* = 12.8 Hz, 1H), 3.06 (d, *J* = 10.4 Hz,1H), 1.45 (s, 9H); ^{13}C-NMR (100 MHz, $CDCl_3$): δ 172.3, 155.5, 142.9, 140.2, 137.7, 131.4, 130.6, 130.1,125.6, 123.1, 79.7, 72.1, 60.4, 53.5, 53.0 52.4, 51.1, 30.6, 29.7, 28.4; HRMS (ESI-TOF): *m/z* (M + H)$^+$ calculated for $C_{19}H_{24}BrIN_3O_4$ 563.9989, found 563.9992.

***N-α*-Boc-1-(3-iodobenzyl)-5-iodo-L-histidine methyl ester (7n):** Yield: 85%; ^{1}H-NMR (400 MHz, $CDCl_3$): δ 7.68–7.65 (m, 2H), 7.47 (br.s, 1H), 7.11–7.07 (m, 1H), 6.98 (d, *J* = 7.6 Hz, 1H), 6.03 (d, *J* = 8.4 Hz, 1H) 5.04 (s, 2H), 4.63–4.59 (m, 1H), 3.70 (s, 3H), 3.13 (dd, *J* = 5.2, 10 Hz, 1H), 3.02 (dd, *J* = 4.8, 9.2 Hz, 1H), 1.46 (s, 9H); ^{13}C-NMR (100 MHz, $CDCl_3$): δ 172.3, 162.5, 155.5, 143.0, 140.1, 137.8, 137.3, 135.9, 130.7, 126.2, 94.7, 79.6, 71.9, 53.0, 52.3, 50.8, 36.5, 31.4, 30.6, 29.7, 28.4; HRMS (ESI-TOF): *m/z* (M + H)$^+$ calculated for $C_{19}H_{24}I_2N_3O_4$ 611.9851, found 611.9854.

***N-α*-Boc-1-(2-iodobenzyl)-5-iodo-L-histidine methyl ester (7o):** Yield: 78%; ^{1}H-NMR (400 MHz, $CDCl_3$): δ 7.89 (d, *J* = 8.4 Hz, 1H), 7.66 (s, 1H), 7.36–7.29 (m, 1H), 7.06–7.05 (m, 1H), 6.52 (d, *J* = 8.0 Hz, 1H), 6.05 (d, *J* = 8.4 Hz, 1H) 5.08 (s, 2H), 4.66–4.62 (m, 1H), 3.72 (s, 3H), 3.17 (dd, *J* = 4.8, 10 Hz, 1H), 3.06 (dd, *J* = 4.8, 9.6 Hz, 1H), 1.46 (s, 9H); ^{13}C-NMR (100 MHz, $CDCl_3$): δ 172.3, 155.5, 142.9, 140.2, 139.9, 139.7, 137.8, 129.8, 129.1, 128.9, 128.3, 97.2, 79.6, 72.4, 56.7, 54.4, 53.1, 52.3, 30.6, 29.7, 28.4; HRMS (ESI-TOF): *m/z* (M + H)$^+$ calculated for $C_{19}H_{24}I_2N_3O_4$ 611.9851, found 611.9851.

2.3. Synthesis of His(1-Bzl-5-iodo)-OMe (8a-o, Scheme 2)

Deprotection of Boc group was carried out using 6N methanolic solution of HCl (5 mL) at ambient temperature for 15 min to 2 h. The solvent was completely removed under reduced pressure to obtain the desired products in 95–100% yield.

2.4. Synthesis of Boc-Trp-His(1-Bzl-5-iodo)-OMe (9a-o, Scheme 2)

The reaction was carried out in a pre-dried MW vial (10 mL) equipped with a magnetic stir bar and all the solid regents were weighed under air. NH_2-His(1-Bzl-5-iodo)-OMe (**8a-o**, 1 mmol) was transferred to MW vial and dissolved in DMF (2 mL) followed by addition of DIEA (4 equiv.) and mixture was stirredat ambient temperature for 2 min. Boc-Trp-OH (1.2 equiv.) was added to the reaction mixture followed by DIC (1.2 equiv.) and HOAt (1.2 equiv.). The reaction mixture was then subjected to MW irradiation for 45 min at 40 W with temperature limit of 60 °C. After completion of the reaction, solvent was evaporated under reduced pressure and the crude product was directly subjected to purification using automated flash column chromatography system by gradient run of CH_2Cl_2 (90–98%) and CH_3OH (2–8%) to obtain dipeptides Boc-Trp-His(1-Bzl-5-iodo)-OMe (**9a-o**) in 72–89% yield.

2.5. Synthesis of Trp-His(1-Bzl-5-iodo)-OMe (10a-o)

Peptide **9a-o** was treated with 6 N methanolic HCl at ambient temperature for 15 min. the solvent was removed completely to obtain the desired peptides **10a-o** in 92–99% yield.

Boc-Trp-His(1-benzyl-5-iodo)-OMe (9a): Yield: 80%; ^{1}H-NMR (500 MHz, CD_3OD): δ 9.08–8.97 (m, 1H), 7.53 (d, *J* = 7.5 Hz, 1H), 7.35–7.29 (m, 5H), 7.22–7.19 (m, 2H), 7.07–7.05 (m, 2H), 7.01–6.95 (m, 1H), 5.34 (s, 2H), 4.74–4.65 (m, 1H), 4.31–4.27 (m, 1H), 3.61 (s, 3H), 3.29–3.19 (m, 1H), 3.15–3.05 (m, 2H), 3.01–2.97 (m, 1H), 1.39–1.36 (m, 9H); ^{13}C-NMR (125 MHz, CD_3OD): δ 173.2, 169.9, 156.4, 137.7, 136.4, 135.0, 133.6, 128.9, 128.6, 127.5, 124.5, 123.3, 121.1, 118.4, 117.9, 110.9, 109.3, 79.6, 74.2, 55.8, 53.6, 51.9, 51.0, 29.6, 28.6, 28.4, 27.4; HRMS (ESI-TOF): *m/z* (M + H)$^+$ calculated for $C_{30}H_{35}IN_5O_5$ 672.1677, found 672.1703; HPLC: t_R = 26.8 min, purity: 96.8%.

Boc-Trp-His[1-(4-*tert*-butylbenzyl)-5-iodo]-OMe (9b): Yield: 88%; ^{1}H-NMR (500 MHz, CD_3OD): δ 7.56–7.52 (m, 1H), 7.30 (d, *J* = 6.5 Hz, 3H), 7.05–6.98 (m, 6H), 5.07 (d, *J* = 11.5 Hz, 1H), 4.70–4.67 (m, 1H), 4.36–4.35 (m, 1H), 3.59 (s, 3H), 3.18–3.15 (m, 1H), 3.08–2.95 (m, 3H), 2.71 (s, 1H), 2.58 (s, 1H), 1.39–1.20 (m, 18H); ^{13}C-NMR (125 MHz, CD_3OD): δ 173.4, 171.3, 156.2, 150.9, 141.0, 140.1, 136.6, 133.0, 127.5, 126.7, 125.5, 123.3, 121.2, 118.6, 118.5, 117.9, 111.0, 109.7, 109.4, 79.2, 73.3, 55.6, 52.2, 51.3, 41.4, 36.1, 34.8, 34.0, 30.4, 28.5, 27.4, 22.2; HRMS (ESI-TOF): *m/z* $(M + H)^+$ calculated for $C_{34}H_{43}IN_5O_5$ 728.2303, found 728.2309; HPLC: t_R = 28.5 min, purity: 95.6%.

Boc-Trp-His[1-(4-*iso*-propylbenzyl)-5-iodo]-OMe (9c): Yield: 82%; ^{1}H-NMR (500 MHz, CD_3OD): δ 7.93 (s, 1H), 7.56 (d, *J* = 6.5 Hz, 1H), 7.29 (d, *J* = 8 Hz, 1H), 7.14 (d, *J* = 8 Hz, 2H), 7.05 (s, 2H), 7.01–6.96 (m, 3H), 5.10 (s, 2H), 4.69–4.67 (m, 1H), 4.36–4.34 (m, 1H), 3.58 (s, 3H), 3.16 (dd, *J* = 5.5, 9 Hz, 1H), 2.98–2.95 (m, 2H), 2.81–2.76 (m, 1H), 1.36–1.26 (m, 9H), 1.14 (d, *J* = 7.0 Hz, 6H); ^{13}C-NMR (125 MHz, CD_3OD): δ 173.1, 171.4, 158.6, 156.1, 148.6, 141.4, 140.2, 138.8, 136.7, 133.6, 127.6, 126.9, 126.6, 123.3, 121.0, 118.5, 118.1, 115.5, 113.4, 110.9, 109.7, 79.3, 73.0, 65.6, 41.4, 33.7, 29.5, 27.4, 23.0, 22.3, 14.2, 13.2; HRMS (ESI-TOF): *m/z* $(M + H)^+$ calculated for $C_{33}H_{41}IN_5O_5$ 714.2147, found 714.2176; HPLC: t_R = 26.3 min, purity: 96.6%.

Boc-Trp-His[1-(3,5-di-*tert*-butylbenzyl)-5-iodo]-OMe (9d): Yield: 72%; ^{1}H-NMR (500 MHz, CD_3OD): δ 7.88 (s, 1H), 7.57 (d, *J* = 7.5 Hz, 1H), 7.34 (s, 1H), 7.29 (d, *J* = 8 Hz, 1H), 7.06–6.97 (m, 5H), 5.09 (s, 2H), 4.66–4.64 (m, 1H), 4.38–4.37 (m, 1H), 3.54 (s, 3H), 3.20 (dd, *J* = 5, 9.5 Hz, 1H), 3.06–2.99 (m, 1H), 2.96–2.93 (m, 2H), 1.32 (s, 6H), 1.25 (s, 21H); ^{13}C-NMR (125 MHz, CD_3OD): δ 173.9, 171.2, 158.6, 156.1, 151.3, 141.2, 140.0, 136.7, 135.2, 121.6, 121.5, 121.0, 118.5, 118.1, 110.9, 109.7, 79.3, 73.1, 55.5, 52.4, 51.8, 51.6, 41.4, 34.4, 30.5, 30.1, 29.4, 28.0, 27.4, 26.9, 22.3; HRMS (ESI-TOF): *m/z* $(M + H)^+$ calculated for $C_{38}H_{51}IN_5O_5$ 784.2929, found 784.2942; HPLC: t_R = 30.9 min, purity: 93.5%.

Boc-Trp-His[1-(3-trifluoromethylbenzyl)-5-iodo]-OMe (9e): Yield: 80%; ^{1}H-NMR (500 MHz,CD_3OD): δ 7.94 (s, 1H), 7.57–7.52 (m, 2H), 7.47–7.43 (m, 2H), 7.29 (d, *J* = 8 Hz, 1H), 7.19 (d, *J* = 7 Hz, 1H), 7.06–7.04 (m, 2H), 7.01–6.94 (m, 1H), 5.21 (s, 2H), 4.68 (t, *J* = 6 Hz, 1H), 4.39–4.33 (m, 1H), 3.60–3.57 (m, 3H), 3.19 (dd, *J* = 5, 9.5 Hz, 1H), 3.02–2.91 (m, 3H), 1.31–1.24 (m, 9H); ^{13}C-NMR (125 MHz, CD_3OD): δ 173.0, 171.3, 158.6, 156.1, 141.9, 140.5, 137.8, 136.7, 130.4, 129.5, 127.6, 125.2, 124.4, 123.5, 121.0, 118.5, 118.1, 110.9, 109.7, 79.3, 72.8, 51.6, 50.5, 41.4, 30.2, 29.4, 28.1, 27.4, 26.9, 22.3; HRMS (ESI-TOF): *m/z* $(M + H)^+$ calculated for $C_{31}H_{34}F_3IN_5O_5$ 740.1551, found 740.1578; HPLC: t_R = 27.6 min, purity: 96.9%.

Boc-Trp-His[1-(2-trifluoromethylbenzyl)-5-iodo]-OMe (9f): Yield: 77%; ^{1}H-NMR (500 MHz,CD_3OD): δ 7.96 (s, 1H), 7.72 (d, *J* = 8.0 Hz, 1H), 7.56–7.49 (m, 2H), 7.42 (t, *J* = 7.5 Hz, 1H), 7.29 (d, *J* = 7.5 Hz, 1H), 7.07–6.97 (m, 4H), 6.46 (d, *J* = 8.0 Hz, 1H), 5.39 (s, 2H), 4.72 (t, *J* = 6.0 Hz, 1H), 4.38 (br.s, 1H), 3.63 (s, 3H), 3.22–3.18 (m, 1H), 3.09–2.92 (m, 3H), 1.39–1.26 (m, 9H); ^{13}C-NMR (125 MHz, CD_3OD): δ 173.0, 171.2, 158.6, 156.1, 140.9, 136.7, 134.8, 132.8, 127.9, 127.1, 125.9, 123.3, 121.0, 118.4, 118.1, 110.9, 109.6, 79.3, 73.6, 55.5, 51.6, 41.4, 30.2, 29.4, 28.1, 27.3, 22.2; HRMS (ESI-TOF): *m/z* $(M + H)^+$ calculated for $C_{31}H_{34}F_3IN_5O_5$ 740.1551, found 740.1563; HPLC: t_R = 28.5 min, purity: 97.6%.

Boc-Trp-His[1-(4-trifluoromethylbenzyl)-5-iodo]-OMe (9g): Yield: 80%; ^{1}H-NMR (500 MHz, CD_3OD): δ 7.99 (s, 1H), 7.58 (br.s, 3H), 7.28 (br.s, 1H), 7.18 (br.s, 2H), 7.06 (br.s, 2H), 7.00–6.97 (m, 1H), 5.25 (s, 1H), 4.69–4.68 (m, 1H), 4.36 (br.s, 1H), 3.59 (s, 3H), 3.19–3.16 (m, 1H), 3.01–2.92 (m, 3H), 1.30 (s, 9H); ^{13}C-NMR (125 MHz, CD_3OD): δ 173.1, 171.3, 158.6, 156.1, 141.6, 140.8, 140.4, 136.7, 129.8, 129.6, 127.2, 125.4, 123.3, 121.0, 118.4, 118.1, 110.9, 109.6, 79.3, 73.3, 51.6, 50.6, 41.4, 30.1, 29.4, 28.1, 27.3, 26.9, 22.2; HRMS (ESI-TOF): *m/z* $(M + H)^+$ calculated for $C_{31}H_{34}F_3IN_5O_5$ 740.1551, found 740.1561; HPLC: t_R = 27.8 min, purity: 96.5%.

Boc-Trp-His[1-(4-nitrobenzyl)-5-iodo]-OMe (9h): Yield: 89%; ^{1}H-NMR (500 MHz, CD_3OD): δ 8.16- 8.11 (m, 2H), 8.06 (s, 1H), 7.53 (d, *J* = 8 Hz, 1H), 7.28 (d, *J* = 7.5 Hz, 1H), 7.19 (d, *J* = 8.5 Hz, 2H), 7.06 (s, 2H), 6.98–6.92 (m, 1H), 5.28 (s, 2H), 4.71 (t, *J* = 6.5 Hz, 1H), 4.36–4.32 (m, 1H), 3.61 (s, 3H), 3.14 (dd, *J* = 5, 9.5 Hz, 1H), 3.04–2.90 (m, 3H), 1.38–1.24 (m, 9H); ^{13}C-NMR (125 MHz, CD_3OD): δ 173.1, 171.2, 156.2, 147.6, 143.6, 141.5, 140.4, 136.7, 127.6, 123.6, 121.1, 118.5, 118.1, 110.9, 109.7, 79.3, 73.5, 52.2, 51.7, 50.5, 30.1, 29.4, 28.1, 27.3,

26.9, 22.2; HRMS (ESI-TOF): *m/z* (M + H)+ calculated for $C_{32}H_{34}IN_6O_7$ 717.1528, found 717.1545; HPLC: t_R = 24.9 min, purity: 89.0%.

Boc-Trp-His[1-(3,4-difluorobenzyl)-5-iodo]-OMe (9i): Yield: 79%; ^{1}H-NMR (500 MHz, CD_3OD): δ 8.12–8.04 (m, 1H), 7.55 (d, *J* = 8 Hz, 1H), 7.30 (d, *J* = 4.5 Hz, 1H), 7.22–7.15 (m, 1H), 7.06–6.99 (m, 5H), 6.84 (d, *J* = 7 Hz, 1H), 5.23–5.12 (m, 2H), 4.70–4.67 (m, 1H), 4.36–4.33 (m, 1H), 3.61 (d, *J* = 10 Hz, 3H), 3.24–3.09 (m, 2H), 3.08–2.88 (m, 4H), 1.32–1.26 (m, 9H); ^{13}C-NMR (125 MHz, CD_3OD): δ 173.6, 171.1, 156.2, 140.8, 140.7, 139.9, 136.7, 133.3, 127.6, 125.9, 123.4, 121.0, 118.5, 118.1, 117.6, 116.1, 110.9, 109.6, 79.4, 73.7, 55.6, 52.1, 51.6, 50.4, 41.4, 35.8, 34.9, 29.9, 29.4, 27.8, 27.3, 26.8, 22.2; HRMS (ESI-TOF): *m/z* (M + H)+ calculated for $C_{30}H_{32}F_2IN_5O_5$ 708.1489, found 708.1501; HPLC: t_R = 26.5 min, purity: 98.8%.

Boc-Trp-His[1-(3,5-difluorobenzyl)-5-iodo]-OMe (9j): Yield: 82%; ^{1}H-NMR (500 MHz, CD_3OD): δ 7.55 (d, *J* = 8.0 Hz, 1H), 7.30–7.29 (m, 1H), 7.06–6.97 (m, 5H), 6.86–6.82 (m, 1H), 6.67 (d, *J* = 4.5 Hz, 1H), 5.28–7.17 (m, 2H), 4.71–4.68 (m, 1H), 4.42–4.33 (m, 1H), 3.61 (d, *J* = 12.0 Hz, 3H), 3.22–3.16 (m, 1H), 3.15–2.93 (m, 3H), 1.36–1.33 (m, 9H); ^{13}C-NMR (125 MHz, CD_3OD): δ 173.4, 170.8, 164.5, 162.0, 158.6, 156.2, 139.9, 136.7, 127.4, 123.3, 121.1, 118.5, 118.1, 117.9, 110.9, 109.9, 109.6, 102.9, 79.4, 51.9, 51.6, 51.2, 50.7, 41.4, 29.8, 29.4, 27.9, 27.4, 22.2; HRMS (ESI-TOF): *m/z* (M + H)+ calculated for $C_{30}H_{33}F_2IN_5O_5$ 708.1489, found 708.1499; HPLC: t_R = 26.0 min, purity: 95.9%.

Boc-Trp-His[1-(3-fluorobenzyl)-5-iodo]-OMe (9k): Yield: 82%; ^{1}H-NMR (500 MHz, CD_3OD): δ 8.07(s, 1H), 7.55 (d, *J* = 8 Hz, 1H), 7.30 (d, *J* = 7.5 Hz, 2H), 7.06 (s, 1H), 6.98 (br.s, 2H), 6.86 (d, *J* = 7.5 Hz, 1H), 6.80 (d, *J* = 9.5 Hz, 1H), 5.19 (s, 2H), 4.69 (t, *J* = 6.5 Hz, 1H), 4.37–4.34 (m, 1H), 3.59 (s, 3H), 3.17 (dd, *J* = 5.5, 9 Hz, 1H), 3.02–2.92 (m, 3H), 1.36–1.24 (m, 9H); ^{13}C-NMR (125 MHz, CD_3OD): δ 173.1, 171.2, 164.1, 162.1, 158.6, 156.2, 140.9, 140.1, 138.8, 136.7, 130.5, 130.5, 127.6, 123.3, 122.5, 121.0, 118.4, 118.1, 114.5, 114.4, 113.7, 113.5, 110.9, 109.6, 79.3, 73.7, 52.1, 51.6, 50.8, 41.4, 29.9, 29.4, 28.0, 27.3, 26.9, 22.2; HRMS (ESI-TOF): *m/z* (M + H)+ calculated for $C_{30}H_{34}FIN_5O_5$ 690.1583, found 690.1599; HPLC: t_R = 25.3 min, purity: 90.4%.

Boc-Trp-His[1-(3-chlorobenzyl)-5-iodo]-OMe (9l): Yield: 77%; ^{1}H-NMR (500 MHz, CD_3OD): δ 7.58- 7.54 (m, 1H), 7.33–7.29 (m, 3H), 7.21 (d, *J* = 12.5 Hz, 1H), 7.06–6.98 (m, 6H), 5.29 (d, *J* = 23 Hz, 2H), 4.71–4.69 (m, 1H), 4.33–4.30 (m, 1H), 3.61 (s, 3H), 3.14–2.94 (m, 6H), 2.79 (s, 1H), 1.40–1.34 (m, 9H); ^{13}C-NMR (125 MHz, CD_3OD): δ 173.4, 170.2, 158.7, 156.2, 139.1, 136.7, 130.3, 128.2, 127.5, 127.2, 125.5, 123.3, 121.1, 118.4, 118.0, 117.9, 110.9, 109.4, 100.7, 79.4, 75.4, 55.7, 51.8, 41.4, 37.5, 36.1, 35.4,34.8, 28.7, 27.9, 27.4, 22.2; HRMS (ESI-TOF): *m/z* (M + H)+ calculated for $C_{30}H_{34}ClIN_5O_5$ 706.1288, found 706.1299; HPLC: t_R = 23.9 min, purity: 97.2%.

Boc-Trp-His[1-(3-bromobenzyl)-5-iodo]-OMe (9m): Yield: 75%; ^{1}H-NMR (500 MHz, CD_3OD): δ 8.06–7.94 (m, 1H), 7.56–7.52 (m, 1H), 7.45–7.37 (m, 1H), 7.31–7.26 (m, 2H), 7.19 (m, 1H), 7.06 (br.s, 2H), 7.01–6.88 (m, 2H), 5.25–5.16 (m, 2H), 4.69–4.67 (m, 1H), 4.35–4.34 (m, 1H), 3.59 (s, 3H), 3.18–3.13 (m, 2H), 3.08–2.95 (m, 2H), 1.31 (s, 9H); ^{13}C-NMR (125 MHz, CD_3OD): δ 172.9, 171.2, 158.6, 156.2, 140.2, 138.6, 130.8, 130.4, 129.8, 125.6, 123.4, 122.4, 121.2, 118.5, 118.1, 111.1, 109.7, 79.4, 73.6, 55.6, 54.5, 52.2, 51.7, 42.5, 41.4, 37.6, 36.1, 35.8, 34.9, 30.0, 28.0, 27.4, 22.2; HRMS (ESI-TOF): *m/z* (M + H)+ calculated for $C_{30}H_{33}IBrN_5O_5$ 750.0783, found 750.0799; HPLC: t_R = 25.5 min, purity: 96.6%.

Boc-Trp-His[1-(3-iodobenzyl)-5-iodo]-OMe (9n): Yield: 81%; ^{1}H-NMR (500 MHz, CD_3OD): δ 8.03- 7.93 (m, 1H), 7.57–7.54 (m, 2H), 7.47 (s, 1H), 7.29 (d, *J* = 6.4 Hz, 1H), 7.07–6.98 (m, 4H), 5.09 (s, 2H), 4.68 (t, *J* = 6.5 Hz, 1H), 4.38–4.33 (m, 1H), 3.59 (s, 3H), 3.19–3.11 (m, 2H), 3.02–2.93 (m, 3H), 1.32 (s, 9H); ^{13}C-NMR (125 MHz, CD_3OD): δ 173.9, 173.1, 171.2, 158.6, 156.1, 140.2, 138.5, 136.9, 135.8, 130.4, 126.2, 123.4, 121.1, 118.5, 110.9, 109.7, 93.9, 79.4, 54.5, 51.7, 48.2, 48.1, 41.4, 38.9, 37.6, 30.0, 27.4, 22.3, 19.5; HRMS (ESI-TOF): *m/z* (M + H)+ calculated for $C_{30}H_{34}I_2N_5O_5$ 798.0644, found 798.0655; HPLC: t_R = 27.6 min, purity: 87.7%.

Boc-Trp-His[1-(2-iodobenzyl)-5-iodo]-OMe (9o): Yield: 76%; ^{1}H-NMR (500 MHz, CD_3OD): δ 7.84 (d, *J* = 8.0 Hz, 1H), 7.77 (s, 1H), 7.56 (d, *J* = 8.0 Hz, 1H), 7.29–7.24 (m, 2H), 7.06–7.04 (m, 2H), 6.97 (t, *J* = 7.5Hz, 2H), 6.40 (d, *J* = 7.5 Hz, 1H), 5.06 (s, 2H), 4.72–7.69 (m, 1H), 4.39–4.37 (m, 1H), 3.63 (d, *J* = 11 Hz, 3H), 3.20 (dd, *J* = 5.0, 9.5 Hz, 1H), 3.04–2.93 (m,

3H), 1.38–1.26 (m, 9H); ^{13}C-NMR (125 MHz, CD_3OD): δ 173.0, 171.3, 163.4, 156.1, 141.8, 140.5, 139.5, 138.2, 136.7, 129.5, 128.7, 127.6, 121.0, 118.5, 110.9, 109.7, 96.5, 79.3, 73.4, 56.4, 55.5, 52.3, 51.7, 41.4, 35.7, 30.3, 29.5, 28.1, 27.4, 26.9, 22.3; HRMS (ESI-TOF): *m*/*z* $(M + H)^+$ calculated for $C_{30}H_{34}I_2N_5O_5$ 798.0644, found 798.0662; HPLC: t_R = 27.5 min, purity: 92.1%.

Trp-His[1-(4-*tert*-butylbenzyl)-5-iodo]-OMe (10b): Yield: 98%; ^{1}H-NMR (500 MHz, CD_3OD): δ 9.16 (s, 1H), 7.64 (d, *J* = 8.0 Hz, 1H), 7.49 (d, *J* = 7.5 Hz, 1H), 7.39–7.35 (m, 2H), 7.23–7.19 (m, 2H), 7.13–7.08 (m, 2H), 7.05–7.02 (m, 2H), 5.34 (s, 2H), 4.69 (t, *J* = 7.0 Hz, 1H), 4.59–4.57 (m, 1H), 4.24–4.21 (m, 1H), 3.60 (s, 3H), 3.39–3.31 (m, 2H), 3.27–3.21 (m, 2H), 3.16–3.12 (m, 1H), 1.29–1.23 (m, 9H); ^{13}C-NMR (125 MHz, CD_3OD): δ 169.7, 168.9, 152.0, 137.5, 136.9, 136.8, 134.2, 130.5, 127.6, 127.0, 125.9, 124.5, 124.2, 121.6, 121.5, 118.9, 117.8, 117.5, 111.4, 111.3, 106.7, 106.4, 79.7, 53.7, 53.6, 52.2, 51.5, 50.8, 41.5, 35.9, 34.8, 35.1, 30.3, 28.0, 27.4, 27.2, 22.2; HRMS (ESI-TOF): *m*/*z* $(M + H)^+$ calculated for $C_{29}H_{35}IN_5O_3$ 628.1779, found 628.1803; HPLC: t_R = 25.5 min, purity: 96.6%.

Trp-His[1-(4-*iso*-propylbenzyl)-5-iodo]-OMe (10c): Yield: 95%; ^{1}H-NMR (500 MHz, CD_3OD): δ 9.14 (s, 1H), 7.63 (d, *J* = 8.0 Hz, 1H), 7.36 (d, *J* = 8.5 Hz, 1H), 7.22–7.19 (m, 5H), 7.11 (t, *J* = 7.5 Hz, 1H), 7.02 (t, *J* = 7.0 Hz, 1H), 5.35 (s, 2H), 4.70 (t, *J* = 7.5 Hz, 1H), 4.22 (dd, *J* = 6.5, 1.5 Hz, 1H), 3.61 (s, 3H), 3.38–3.32 (m, 1H), 3.26–3.19 (m, 2H), 3.16–3.11 (m, 1H), 1.17 (d, *J* = 7.5 Hz, 6H); ^{13}C-NMR (125 MHz, CD_3OD): δ 169.7, 168.9, 149.8, 137.6, 136.9, 134.4, 130.9, 127.9, 126.9, 124.4, 121.5, 118.9, 117.8, 115.5, 111.3, 106.4, 79.4, 71.9, 53.6, 52.1, 51.5, 41.4, 33.7, 28.0, 27.4, 22.9, 22.2; HRMS (ESI-TOF): *m*/*z* $(M + H)^+$ calculated for $C_{28}H_{33}IN_5O_3$ 614.1623, found 614.1643; HPLC: t_R = 23.7 min, purity: 96.9%.

Trp-His[1-(3,5-di-*tert*-butylbenzyl)-5-iodo]-OMe (10d): Yield: 97%; ^{1}H-NMR (500 MHz, CD_3OD): δ 9.19 (s, 1H), 7.63 (d, *J* = 7.5 Hz, 1H), 7.41 (s, 1H), 7.35 (d, *J* = 8.0 Hz, 1H), 7.23 (s, 4H), 7.08 (t, *J* = 7.5 Hz, 1H), 7.00 (t, *J* = 7.5 Hz, 1H), 5.35 (s, 2H), 4.69 (t, *J* = 6.5 Hz, 1H), 4.23 (t, *J* = 7.0 Hz, 1H), 3.56 (s, 3H), 3.39 (dd, *J* = 6.5 Hz, 1H), 3.23 (dd, *J* = 7.5 Hz, 2H), 3.10 (dd, *J* = 6.5 Hz, 1H), 1.28 (s, 18H); ^{13}C-NMR (125 MHz, CD_3OD): δ 169.6, 168.9, 151.9, 137.4, 136.9, 134.2, 132.8, 127.1, 124.5, 122.7, 122.5, 121.5, 118.9, 117.8, 111.3, 106.4, 79.7, 54.4, 53.7, 52.1, 51.5, 41.4, 34.5, 34.1, 30.5, 29.4, 28.1, 27.3, 22.3; HRMS (ESI-TOF): *m*/*z* $(M + H)^+$ calculated for $C_{33}H_{43}IN_5O_3$ 684.2403, found 684.2420; HPLC: t_R = 26.9 min, purity: 97.6%.

Trp-His[1-(3-trifluoromethylbenzyl)-5-iodo]-OMe (10e): Yield: 92%; ^{1}H-NMR (500 MHz, CD_3OD): δ 9.28 (s, 1H), 7.66–7.61 (m, 4H), 7.58–7.55 (m, 1H), 7.49 (d, *J* = 7.5 Hz, 1H), 7.36 (d, *J* = 8.5 Hz, 1H), 7.22 (s, 1H), 7.11–7.08 (m, 1H), 7.02 (t, *J* = 7.5 Hz, 1H), 5.52 (s, 2H), 4.72 (t, *J* = 7 Hz, 1H), 4.23–4.20 (m, 1H), 3.61 (s, 3H), 3.39–3.35 (m, 1H), 3.29–3.2 (m, 2H), 3.12 (dd, *J* = 6.5, 8.5 Hz); ^{13}C-NMR (125 MHz, CD_3OD): δ 169.7, 168.9, 149.9, 138.0, 136.9, 135.0, 134.7, 131.4, 131.2, 130.9, 129.9, 127.0, 125.3, 124.6, 124.5, 122.9, 121.5, 118.9, 117.8, 115.5, 111.3, 106.4, 79.5, 53.7, 53.1, 52.1, 51.5, 41.5, 29.4, 28.1, 27.3, 22.2; HRMS (ESI-TOF): *m*/*z* $(M + H)^+$ calculated for $C_{26}H_{26}F_3IN_5O_3$ 640.1027, found 640.1053; HPLC: t_R = 24.7 min, purity: 94.5%.

Trp-His[1-(2-trifluoromethylbenzyl)-5-iodo]-OMe (10f): Yield: 95%; ^{1}H-NMR (500 MHz, CD_3OD): δ 9.16 (s, 1H), 7.81 (d, *J* = 7.5 Hz, 1H), 7.68–7.48 (m, 4H), 7.36 (d, *J* = 8.5 Hz, 1H), 7.23 (s, 1H), 7.11 (t, *J* = 7 Hz, 1H), 7.06–7.01 (m, 1H), 6.85 (d, *J* = 7.5 Hz, 1H), 5.63 (s, 2H), 4.75 (t, *J* = 6.5 Hz, 1H), 4.26–4.23 (m, 1H), 3.62 (s, 3H), 3.40 (dd, *J* = 6.5, 8.5 Hz, 1H), 3.27–3.23 (m, 1H), 3.16 (dd, *J* = 7.0, 8.0 Hz, 1H), 2.88–2.77 (m, 1H); ^{13}C-NMR (125 MHz, CD_3OD): δ 169.6, 168.9, 158.5, 138.5, 136.9, 134.7, 133.1, 131.7, 128.9, 128.1, 127.0, 126.5, 124.4, 121.5, 118.9, 117.8, 117.5, 111.3, 106.4, 80.0, 53.7, 52.2, 51.5, 50.9, 41.7, 35.8, 29.4, 28.2, 27.4, 22.1, 20.1; HRMS (ESI-TOF): *m*/*z* $(M + H)^+$ calculated for $C_{26}H_{26}F_3IN_5O_3$ 640.1027, found 640.1048; HPLC: t_R = 24.9 min, purity: 96.6%.

Trp-His[1-(4-trifluoromethylbenzyl)-5-iodo]-OMe (10g): Yield: 98%; ^{1}H-NMR (500 MHz, CD_3OD): δ 9.28 (s, 1H), 7.68–7.62 (dd, *J* = 7.5, 11.5 Hz, 4H), 7.42 (d, *J* = 8.0 Hz, 2H), 7.36 (d, *J* = 8.0 Hz, 1H), 7.23–7.22 (m, 1H), 7.10 (t, *J* = 7.5 Hz, 1H), 7.05–7.00 (m, 1H), 5.53 (s, 2H), 4.74–4.71 (m, 1H), 4.27–4.21 (m, 1H), 3.63 (s, 3H), 3.38 (dd, *J* = 6.5, 8.0 Hz, 1H), 3.67–3.21 (m, 2H), 3.19–3.10 (m, 1H); ^{13}C-NMR (125 MHz, CD_3OD): δ 169.7, 168.9, 158.6, 138.1, 136.9, 134.6, 130.6, 128.1, 128.0, 127.0, 125.7, 124.4, 121.5, 118.9, 117.8, 111.3, 106.4, 79.7,

53.7, 53.1, 52.2, 51.5, 41.5, 29.4, 28.2, 27.4, 22.2; HRMS (ESI-TOF): *m/z* (M + H)+ calculated for $C_{26}H_{26}F_3IN_5O_3$ 640.1027, found ; 640.1042; HPLC: t_R = 25.1 min, purity: 91.6%.

Trp-His[1-(4-nitrobenzyl)-5-iodo]-OMe (10h): Yield: 98%; ^{1}H-NMR (500 MHz, CD_3OD): δ 9.26 (s,1H), 8.19 (d, *J* = 8.5 Hz, 2H), 7.67–7.35 (m, 2H), 7.45 (d, *J* = 8.5 Hz, 2H), 7.36 (d, *J* = 8 Hz, 1H), 7.22 (s,1H), 7.11–7.08 (m, 1H), 7.01 (t, *J* = 7.5 Hz, 1H), 5.57 (s, 2H), 4.73–4.71 (m, 1H), 4.24–4.21 (m, 1H), 3.64 (s, 3H), 3.36 (dd, *J* = 6.5, 8.5 Hz, 1H), 3.25–3.19 (m, 2H), 3.13 (dd, *J* = 7, 8 Hz, 1H); ^{13}C-NMR (125 MHz, CD_3OD): δ 169.7, 168.8, 148.0, 140.9, 138.4, 136.9, 135.0, 128.8, 128.5, 127.0, 124.4, 124.0, 123.8, 121.5, 118.9, 117.8, 111.3, 106.4, 79.6, 53.7, 52.8, 52.2, 51.5, 51.0, 29.4, 28.2, 27.4, 19.5; HRMS (ESI-TOF): *m/z* (M + H)+ calculated for $C_{25}H_{26}IN_6O_5$ 617.1004, found 617.1022; HPLC: t_R = 17.8 min, purity: 93.9%.

Trp-His[1-(3,4-difluorobenzyl)-5-iodo]-OMe (10i): Yield: 99%; ^{1}H-NMR (500 MHz, CD_3OD): δ 9.23 (s, 1H), 7.62 (d, *J* = 8.0 Hz, 1H), 7.38–7.35 (m, 2H0, 7.27–7.22 (m, 3H), 7.13–7.08 (m, 2H), 7.05–6.99 (m, 2H), 5.39 (s, 2H), 4.72 (t, *J* = 7.0 Hz, 1H), 4.22 (t, *J* = 7.0 Hz, 1H), 3.62 (s, 3H), 3.43–3.35 (m, 2H), 3.27–3.22 (m, 2H), 3.15–3.09 (m, 1H); ^{13}C-NMR (125 MHz, CD_3OD): δ 169.7, 168.9, 151.5, 149.3, 137.9, 136.9, 134.5, 127.0, 124.5, 121.5, 118.9, 117.9, 117.8, 117.2, 111.4, 106.4, 79.6, 53.7, 52.7, 52.2, 51.4, 41.6, 34.0, 29.4, 28.1, 27.4, 22.1; HRMS (ESI-TOF): *m/z* (M + H)+ calculated for $C_{25}H_{25}F_2IN_5O_3$ 608.0965, found 608.0981; HPLC: t_R = 22.7 min, purity: 97.8%.

Trp-His[1-(3,5-difluorobenzyl)-5-iodo]-OMe (10j): Yield: 96%; ^{1}H-NMR (500 MHz, CD_3OD): δ 9.27 (s, 1H), 7.63–7.61 (m, 1H), 7.38–7.35 (m, 2H), 7.27–7.19 (m, 1H), 7.13–7.08 (m, 1H), 7.05–7.00 (m, 2H), 6.96–6.88 (m, 2H), 5.45 (s, 2H), 4.73 (t, *J* = 6.5 Hz, 1H), 4.31–4.21 (m, 1H), 3.63 (s, 3H), 3.46–3.36 (m, 2H), 3.27–3.23 (m, 2H), 3.14–3.10 (m, 2H); ^{13}C-NMR (125 MHz, CD_3OD): δ 169.6, 168.8, 158.4, 138.2, 136.9, 134.6, 127.0, 124.5, 121.6, 118.9, 117.5, 111.4, 110.8, 106.4, 103.7, 79.8, 53.7, 53.3, 52.7, 52.2,51.5, 29.4, 28.2, 27.3, 26.3, 22.1; HRMS (ESI-TOF): *m/z* (M + H)+ calculated for $C_{25}H_{25}F_2IN_5O_3$ 608.0965, found 608.0987; HPLC: t_R = 22.3 min, purity: 98.9%.

Trp-His[1-(3-fluorobenzyl)-5-iodo]-OMe (10k): Yield: 97%; ^{1}H-NMR (500 MHz, CD_3OD): δ 8.05–7.99 (m, 2H), 7.72 (s, 1H), 7.49–7.46 (m, 1H), 7.43–7.40 (m, 1H), 7.37–7.29 (m, 2H), 7.16–7.09 (m, 2H), 7.05–7.02 (m, 1H), 6.99–6.97 (m, 1H), 5.32 (s, 2H), 4.72–4.69 (m, 1H), 3.79–3.73 (m, 1H), 3.60 (s, 3H), 3.42–3.35 (m, 1H), 3.22–3.05 (m, 3H); ^{13}C-NMR (100 MHz, CD_3OD): δ 170.8, 168.0, 140.9, 136.6, 131.2, 129.9, 124.7, 122.2, 121.3, 119.1, 118.3, 114.4, 111.3, 110.2, 108.5, 106.6, 85.3, 75.4, 51.9, 41.4, 28.6, 27.4, 22.2; HRMS (ESI) m/z (M + H)+ calculated for $C_{25}H_{26}FIN_5O_3$ 590.1059, found 590.1073; HPLC: t_R = 22.7 min, 97.4%.

Trp-His[1-(3-chlorobenzyl)-5-iodo]-OMe (10l): Yield: 95%; ^{1}H-NMR (500 MHz, CD_3OD): δ 9.19 (s,1H), 7.62 (d, *J* = 8 Hz, 1H), 7.58–7.54 (m, 1H), 7.49 (d, *J* = 8 Hz, 1H), 7.39–7.34 (m, 4H), 7.18–7.16 (m,1H), 7.14–7.09 (m, 1H), 7.06–7.01 (m, 2H), 5.41 (s, 2H), 4.73–4.70 (m, 1H), 4.59–4.55 (m, 1H), 3.66 (s, 1H), 3.62 (s, 3H), 3.39–3.35 (m, 1H), 3.24–3.21 (m, 2H), 3.17–3.09 (m, 1H); ^{13}C-NMR (125 MHz, CD_3OD): δ 169.8, 168.8, 136.9, 136.1, 130.5, 128.7, 127.6, 127.0, 125.9, 124.4, 121.5, 118.9, 117.7, 117.5, 111.3, 106.4, 53.7, 52.1, 51.5, 41.4, 35.8, 27.4, 22.2; HRMS (ESI-TOF): *m/z* (M + H)+ calculated for $C_{25}H_{26}ClIN_5O_3$ 606.0763, found 606.0777; HPLC: t_R = 23.6 min, purity: 90.2%.

Trp-His[1-(3-bromobenzyl)-5-iodo]-OMe (10m): Yield: 98%; ^{1}H-NMR (500 MHz, CD_3OD): δ 7.67- 7.61 (m, 1H), 7.49–7.46 (m, 1H), 7.38–7.35 (m, 2H), 7.23–7.18 (m, 3H), 7.13–7.08 (m, 2H), 7.06–7.00 (m, 2H), 5.38 (s, 2H), 4.72–4.69 (m, 1H), 4.25–4.16 (m, 1H), 3.63 (s, 3H), 3.22–3.17 (m, 4H); ^{13}C-NMR (125 MHz, CD_3OD): δ 169.9, 168.8, 158.6, 136.9, 130.7, 127.0, 126.4, 124.2, 121.6, 119.0, 117.5, 111.4, 106.7, 78.2, 77.7, 54.5, 52.7, 42.5, 41.4, 35.9, 34.1, 27.4, 22.3, 17.5, 16.0; HRMS (ESI-TOF): *m/z* (M + H)+ calculated for $C_{25}H_{26}BrIN_5O_3$ 650.0258, found 650.0273; HPLC: t_R = 24.5 min, purity: 96.6%.

Trp-His[1-(3-iodobenzyl)-5-iodo]-OMe (10n): Yield: 96%; ^{1}H-NMR (500 MHz, CD_3OD): δ 9.17 (s,1H), 7.75–7.07 (m, 1H), 7.67–7.61 (m, 2H), 7.59–7.48 (m, 1H), 7.36 (d, *J* = 7.5 Hz, 1H), 7.22–7.16 (m, 2H), 7.13–7.09 (m, 2H), 7.06–6.99 (m, 1H), 5.50–5.31 (m, 2H), 4.72–6.98 (m, 1H), 4.27–4.19 (m, 1H), 3.70–3.67 (m, 1H), 3.62 (s, 3H), 3.42–3.34 (m, 1H), 3.25–3.18 (m, 2H), 3.14–3.09 (m, 1H); ^{13}C-NMR (125 MHz, CD_3OD): δ 169.9, 168.8, 158.6, 138.0, 137.8, 136.6, 136.1, 130.7, 127.0, 124.5, 121.5, 118.9, 117.8, 111.4, 106.4, 94.1, 54.5,

53.7, 52.2, 51.5, 42.5, 41.4, 38.9, 34.0, 27.4, 22.2, 17.43; HRMS (ESI-TOF): *m*/*z* (M + H)$^+$ calculated for $C_{25}H_{26}I_2N_5O_3$ 698.0120, found 698.0138; HPLC: t_R = 25.5 min, purity: 98.0%.

Trp-His[1-(2-iodobenzyl)-5-iodo]-OMe (10o): Yield: 97%; ^{1}H-NMR (500 MHz, CD_3OD): δ 8.93 (s,1H), 7.94 (d, *J* = 7.5 Hz, 1H) 7.63 (d, *J* = 8.0 Hz, 1H), 7.39–7.35 (m, 2H), 7.23 (s, 1H), 7.12–7.08 (m, 2H), 7.02 (t, *J* = 7.5 Hz, 1H), 6.88 (d, *J* = 7.5 Hz, 1H), 5.36 (s, 2H), 4.74 (t, *J* = 6.5 Hz, 1H), 4.24 (t, *J* = 6.5 Hz, 1H), 3.65 (m, 3H), 3.39 (dd, *J* = 6.0, 8.5 Hz, 1H), 3.27–3.23 (m, 2H), 3.17–3.13 (m, 1H); ^{13}C-NMR (125 MHz, CD_3OD): δ 169.7, 168.9, 140.1, 138.1, 136.9, 135.5, 134.6, 130.5, 128.9, 128.9, 127.0, 124.5, 121.5, 118.9, 117.8, 111.3, 106.4, 97.6, 80.0, 58.9, 53.7, 52.3, 51.6, 41.4, 28.2, 27.4, 22.2, 19.2; HRMS (ESI-TOF): *m*/*z* (M + H)$^+$ calculated for $C_{25}H_{26}I_2N_5O_3$ 698.0120, found 698.0135; HPLC: t_R = 26.4 min, purity: 97.9%.

3. NMR Spectra of the Representative Histidine Derivatives and Peptides

3.1. ^{1}H-NMR Spectra

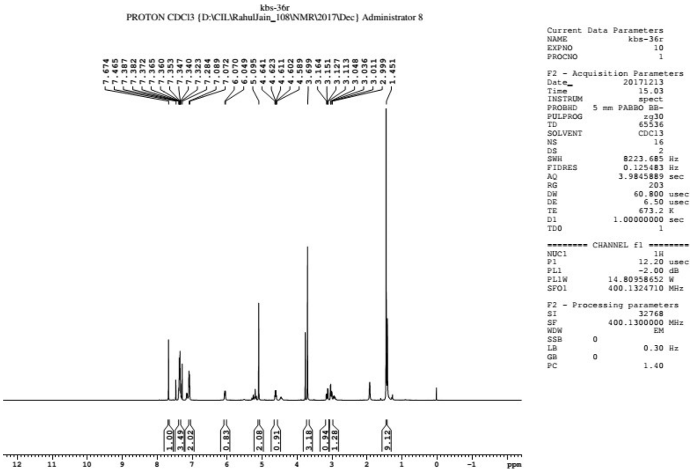

Figure S1. *N*-α-Boc-1-benzyl-5-iodo-L-histidine methyl ester (**7a**).

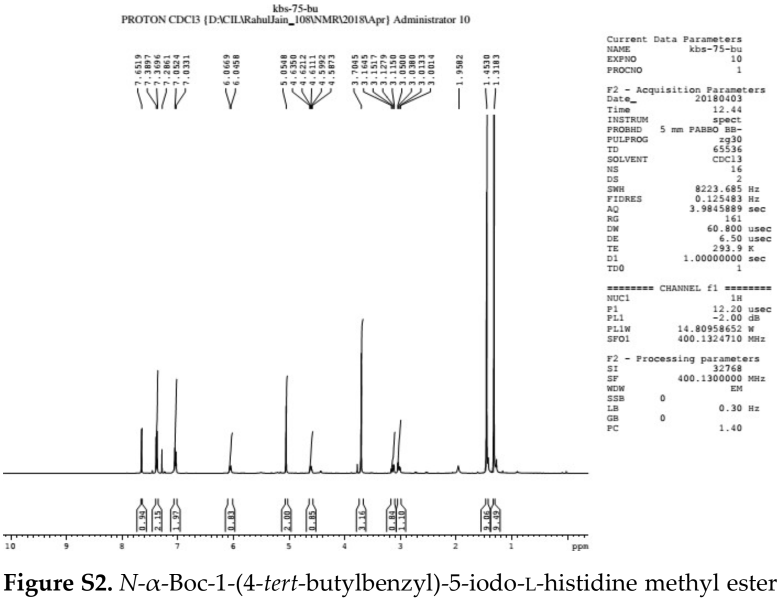

Figure S2. *N*-α-Boc-1-(4-*tert*-butylbenzyl)-5-iodo-L-histidine methyl ester (**7b**).

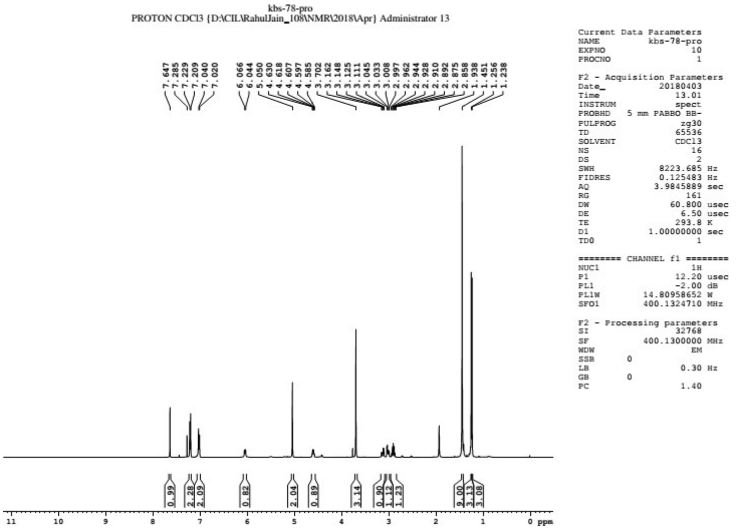

Figure S3. *N*-α-Boc-1-(4-*iso*-propyl)-5-iodo-L-histidine methyl ester (**7c**).

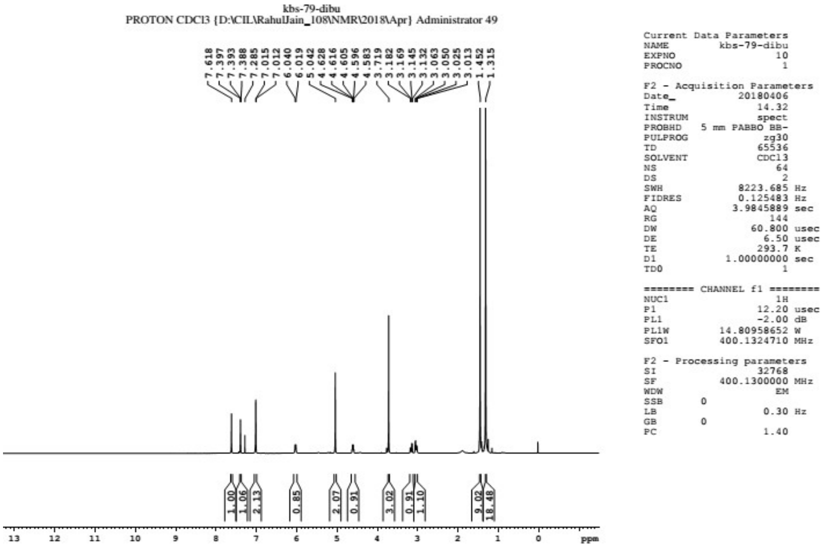

Figure S4. *N*-α-Boc-1-(3,5-di-*tert*-butylbenzyl)-5-iodo-L-histidine methyl ester (**7d**).

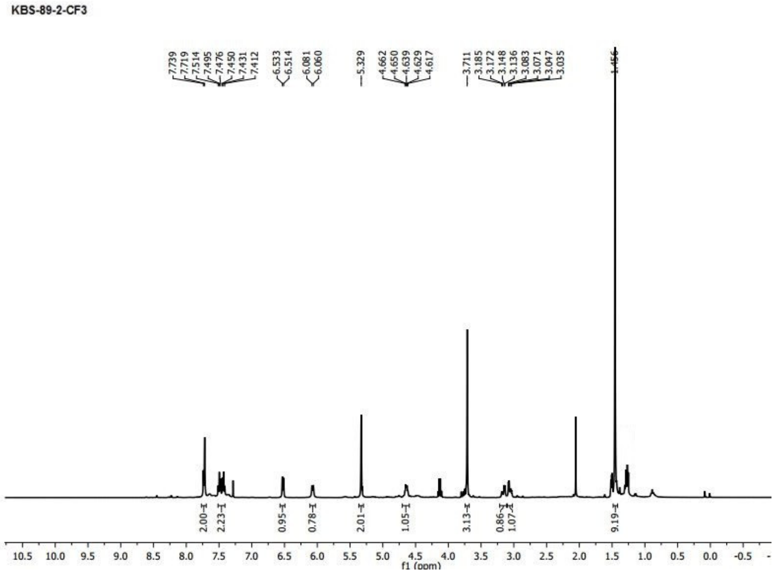

Figure S5. *N*-α-Boc-1-(2-trifluoromethyl)-5-iodo-L-histidine methyl ester (**7f**).

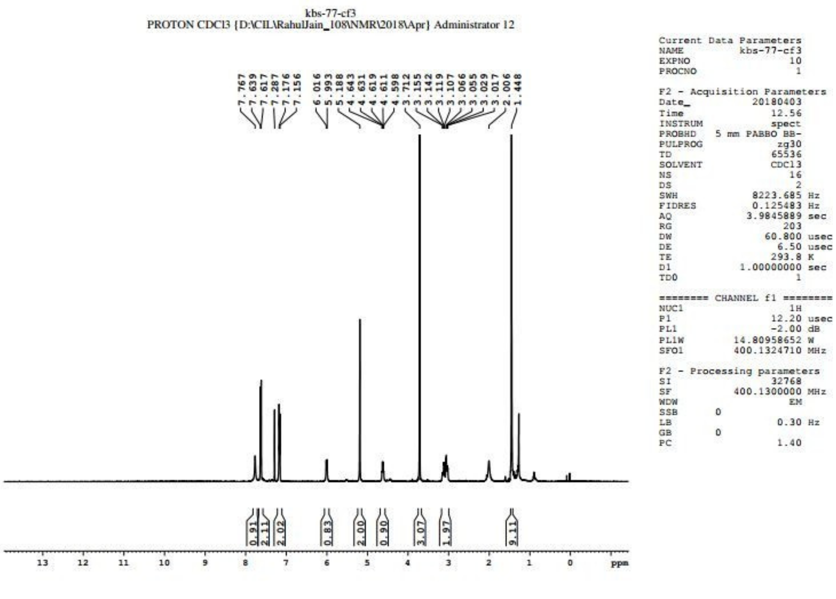

Figure S6. *N*-α-Boc-1-(4-trifluoromethyl)-5-iodo-L-histidine methyl ester (**7g**).

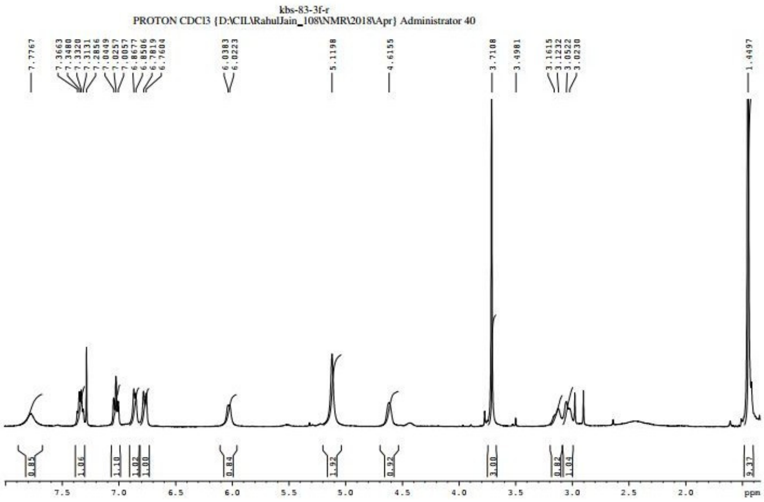

Figure S7. *N*-α-Boc-1-(3-fluoro)-5-iodo-L-histidine methyl ester (**7k**).

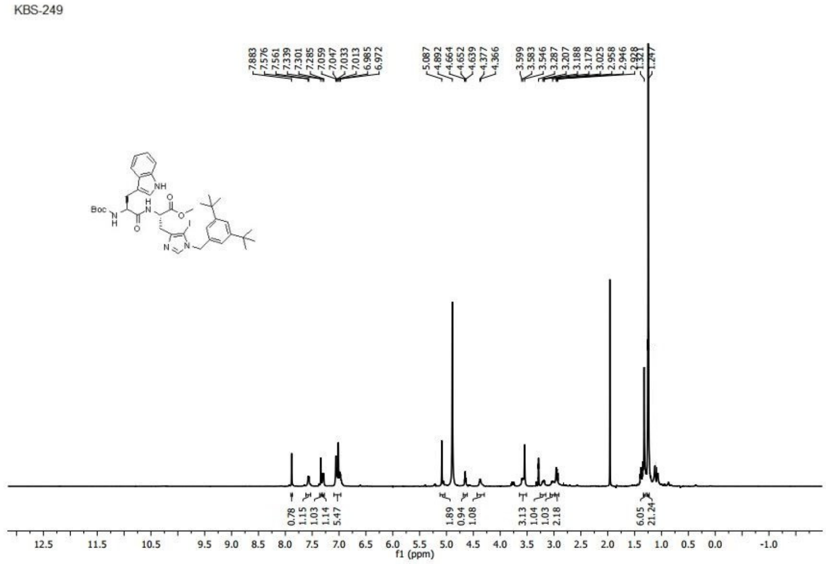

Figure S8. Boc-Trp-His[1-(3,5-di-*tert*-butylbenzyl)-5-iodo]-OMe (**9d**).

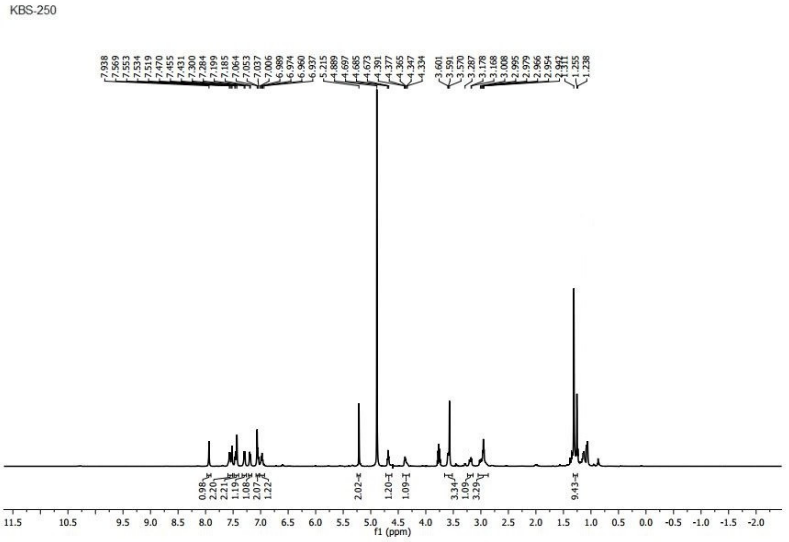

Figure S9. Boc-Trp-His[1-(3-trifluoromethylbenzyl)-5-iodo]-OMe (**9e**).

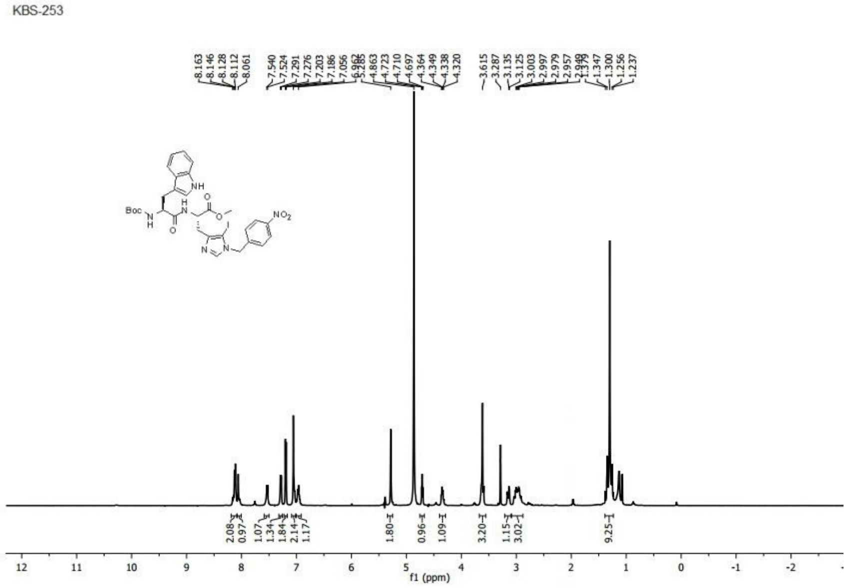

Figure S10. Boc-Trp-His[1-(4-nitrobenzyl)-5-iodo]-OMe (**9h**).

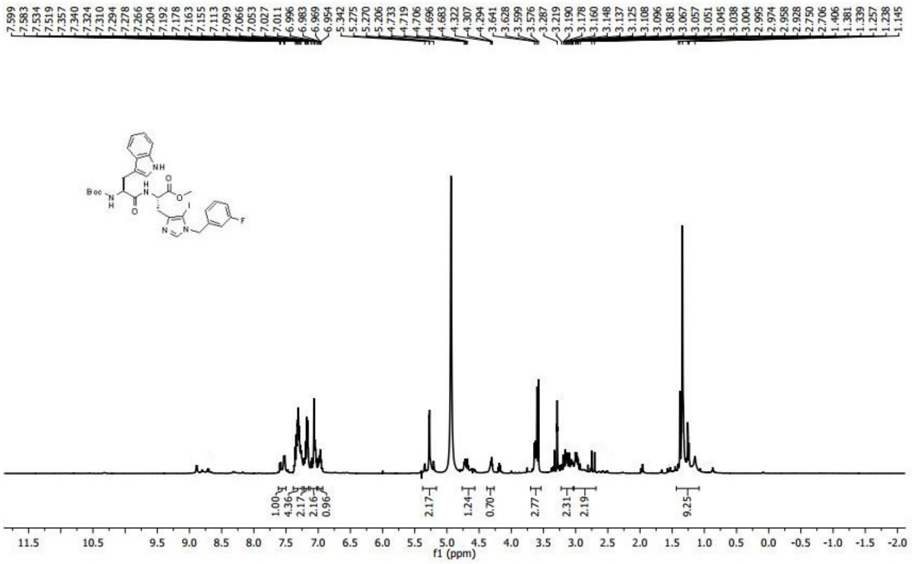

Figure S11. Boc-Trp-His[1-(3-fluorobenzyl)-5-iodo]-OMe (**9k**).

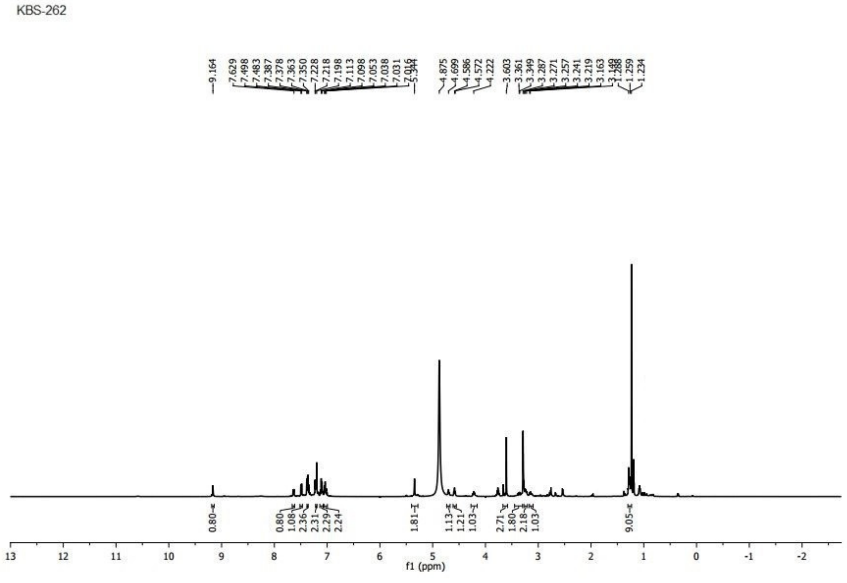

Figure S12. Trp-His[1-(4-*tert*-butylbenzyl)-5-iodo]-OMe (**10b**).

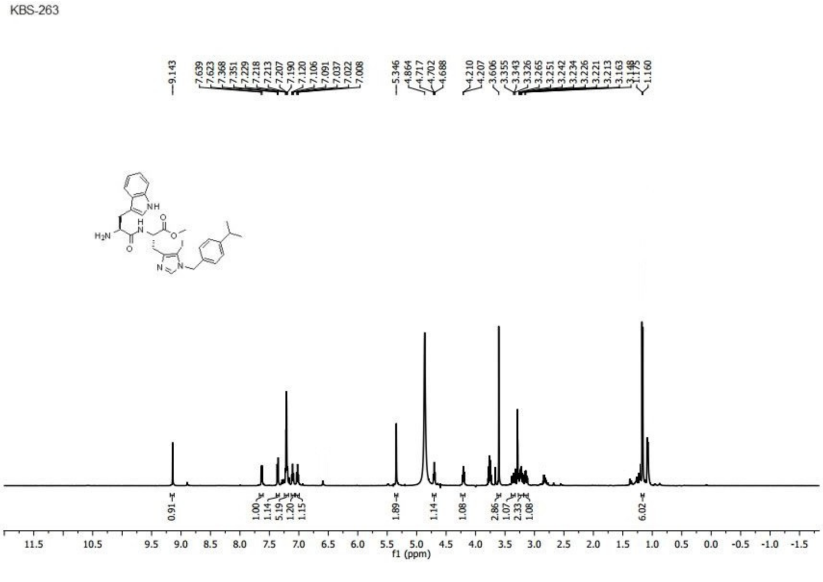

Figure S13. Trp-His[1-(4-*iso*-propylbenzyl)-5-iodo]-OMe (**10c**).

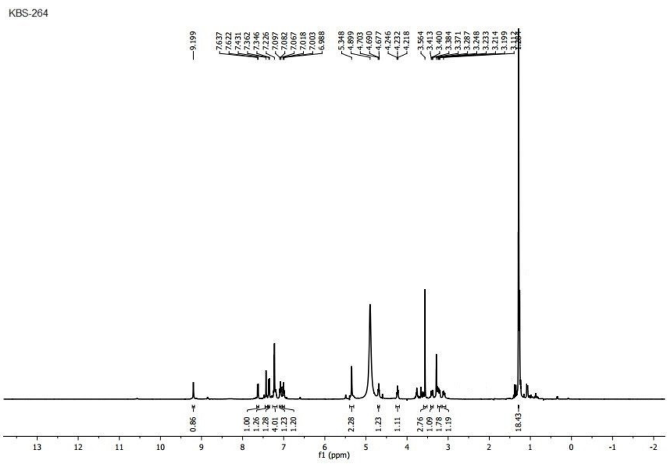

Figure S14. Trp-His[1-(3,5-di-*tert*-butylbenzyl)-5-iodo]-OMe (**10d**).

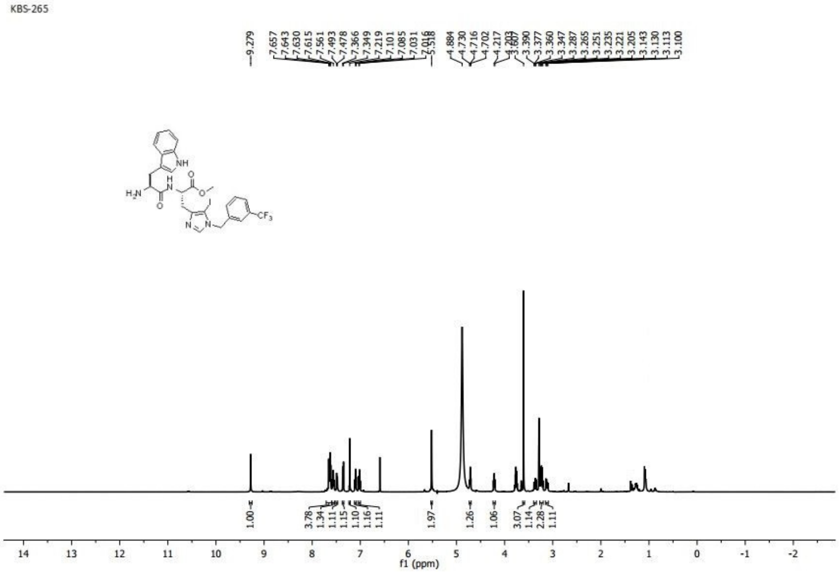

Figure S15. Trp-His[1-(3-trifluoromethylbenzyl)-5-iodo]-OMe (**10e**).

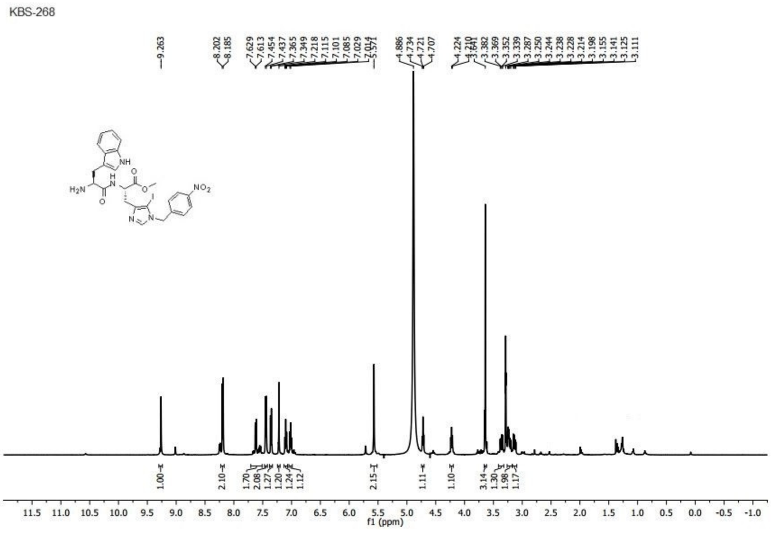

Figure S16. Trp-His[1-(4-nitrobenzyl)-5-iodo]-OMe (**10h**).

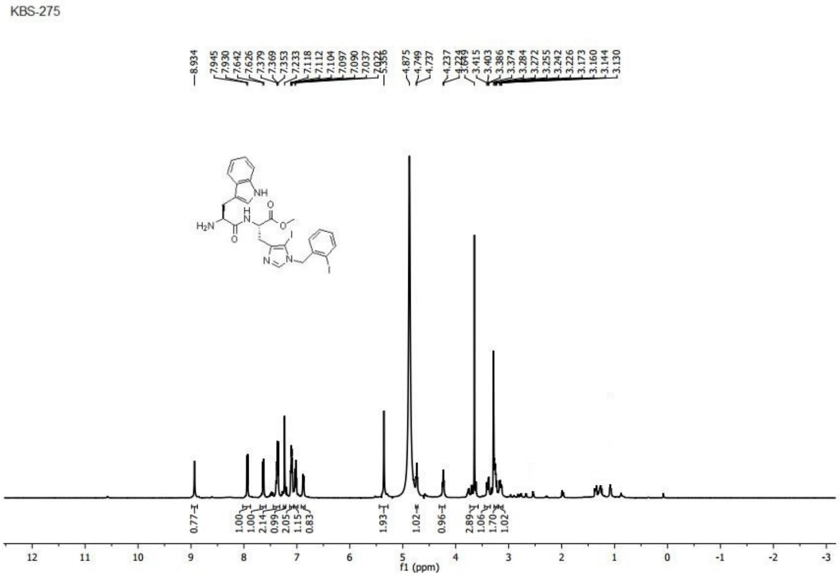

Figure S17. Trp-His[1-(2-iodobenzyl)-5-iodo]-OMe (**10o**).

3.2. ^{13}C-NMR Spectra

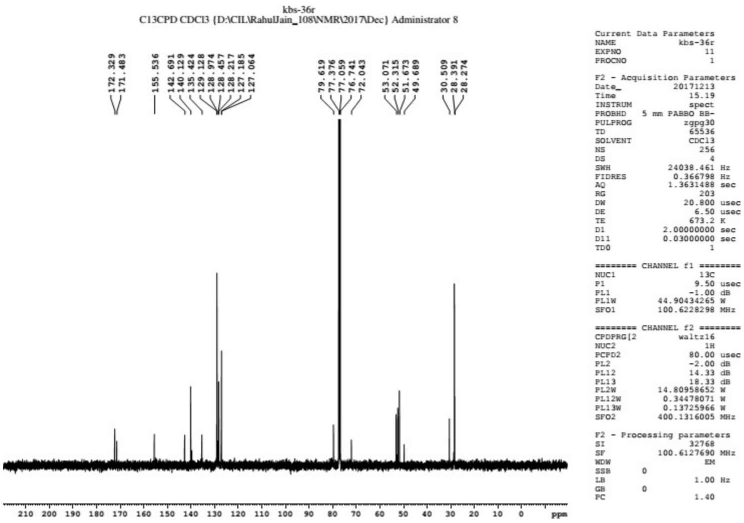

Figure S18. *N*-α-Boc-1-benzyl-5-iodo-L-histidine methyl ester (**7a**).

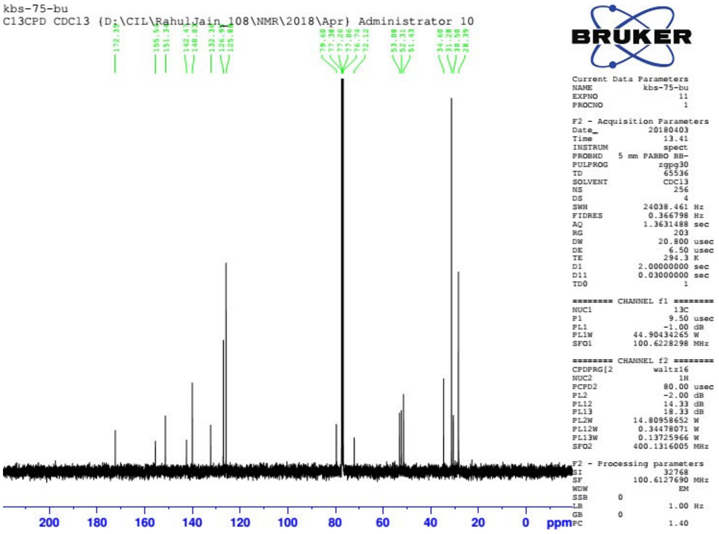

Figure S19. *N*-α-Boc-1-(4-*tert*-butylbenzyl)-5-iodo-L-histidine methyl ester (**7b**).

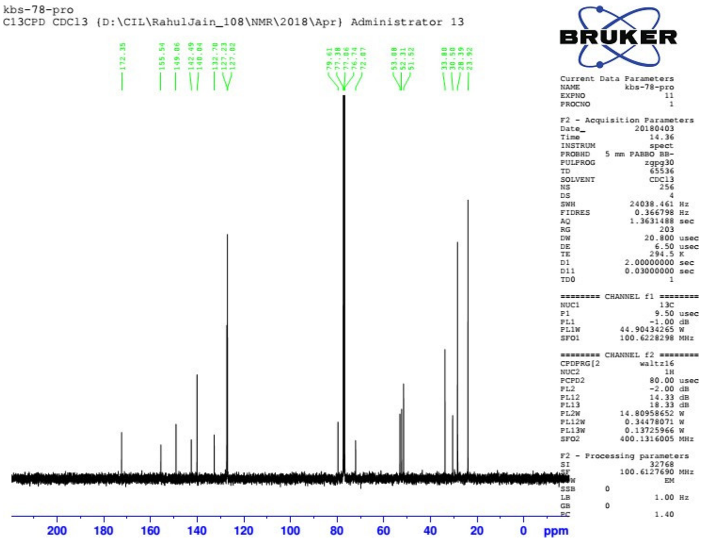

Figure S20. N-α-Boc-1-(4-*iso*-propyl)-5-iodo-L-histidine methyl ester (**7c**).

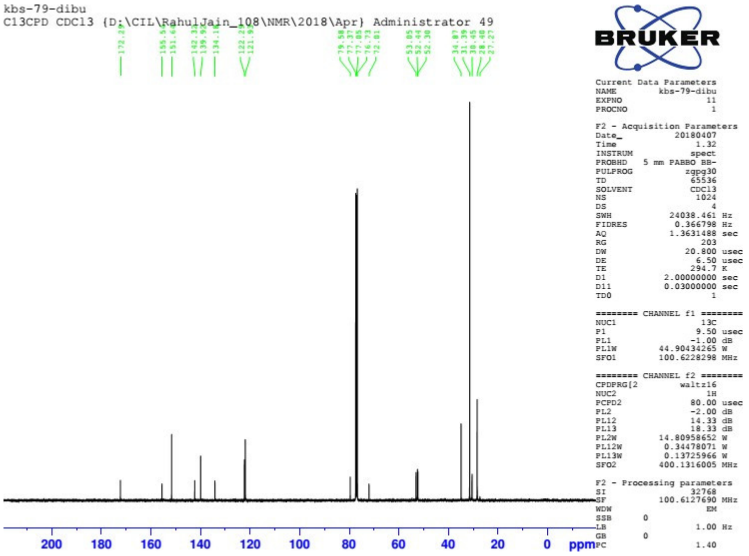

Figure S21. N-α-Boc-1-(3,5-di-*tert*-butylbenzyl)-5-iodo-L-histidine methyl ester (**7d**).

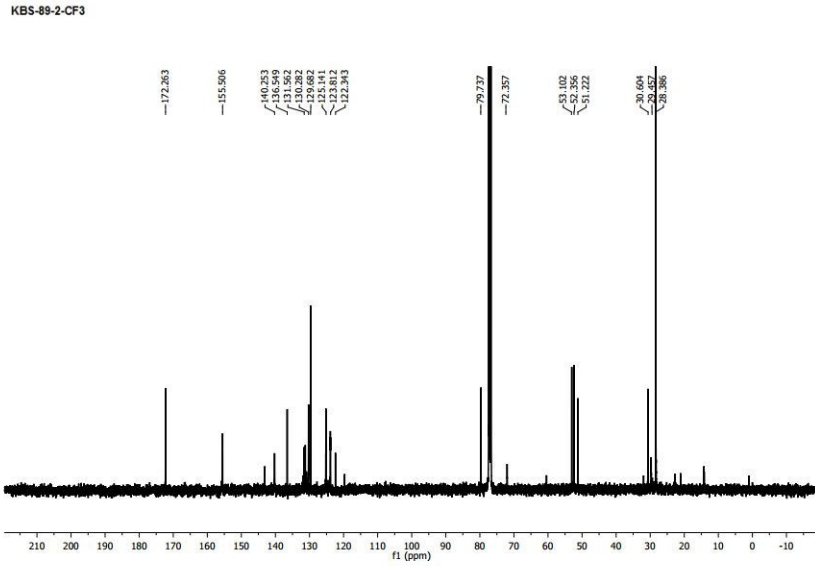

Figure S22. N-α-Boc-1-(2-trifluoromethyl)-5-iodo-L-histidine methyl ester (**7f**).

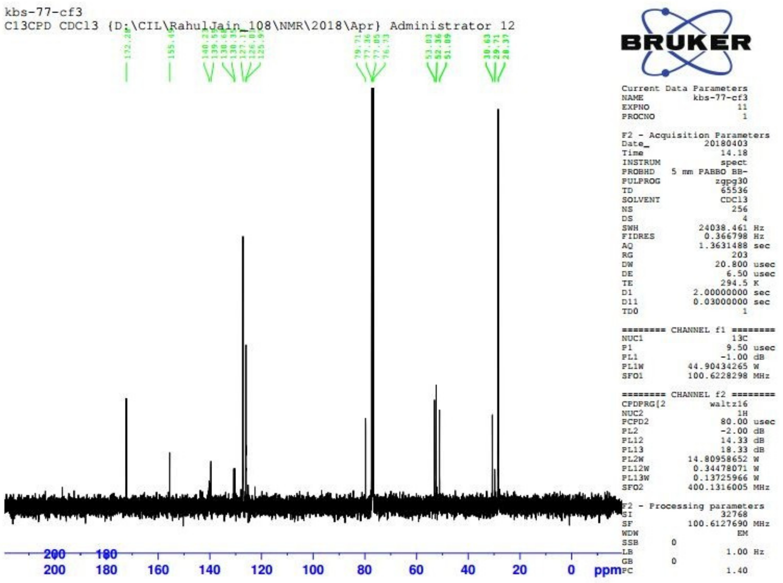

Figure S23. *N*-α-Boc-1-(4-trifluoromethyl)-5-iodo-L-histidine methyl ester (**7g**).

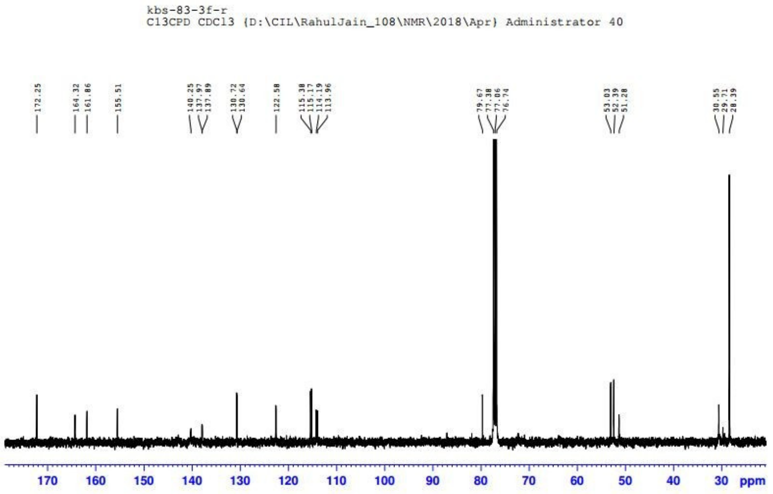

Figure S24. *N*-α-Boc-1-(3-fluoro)-5-iodo-L-histidine methyl ester (**7k**).

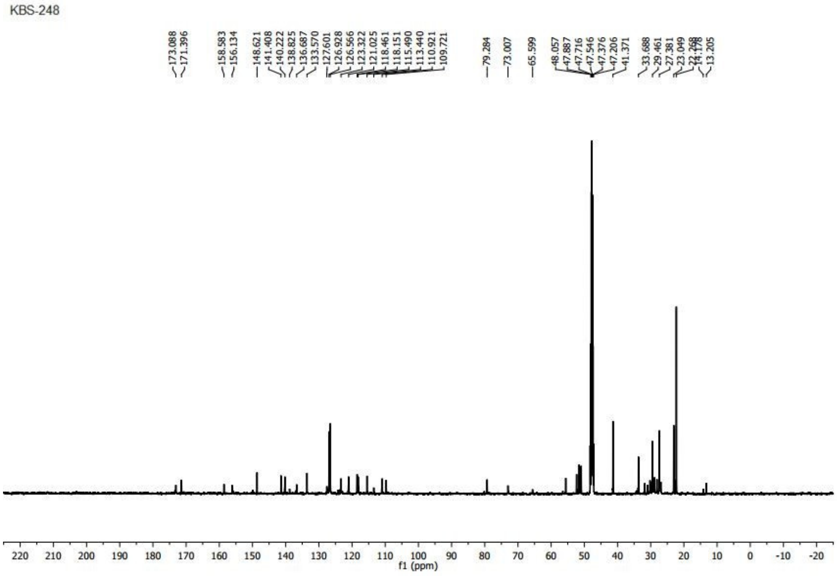

Figure S25. Boc-Trp-His[1-(4-*iso*-propylbenzyl)-5-iodo]-OMe (**9c**).

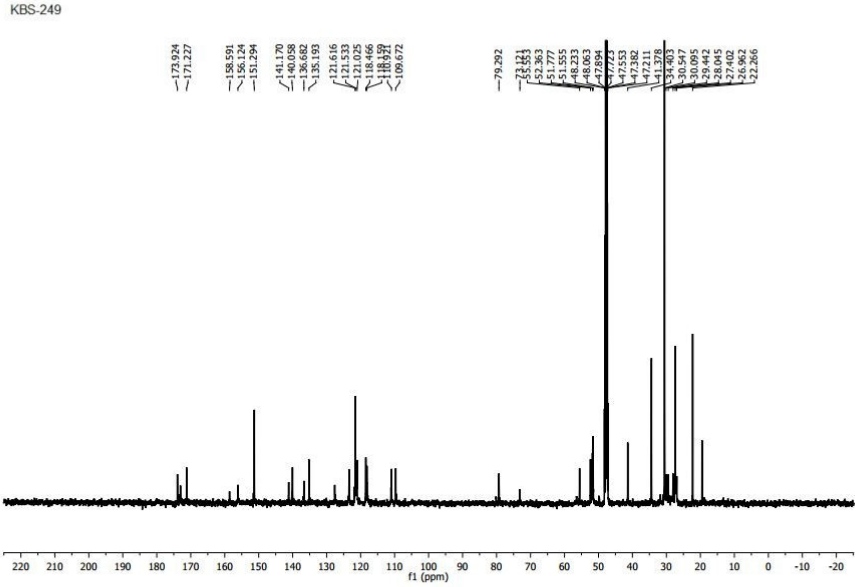

Figure S26. Boc-Trp-His[1-(3,5-di-*tert*-butylbenzyl)-5-iodo]-OMe (**9d**).

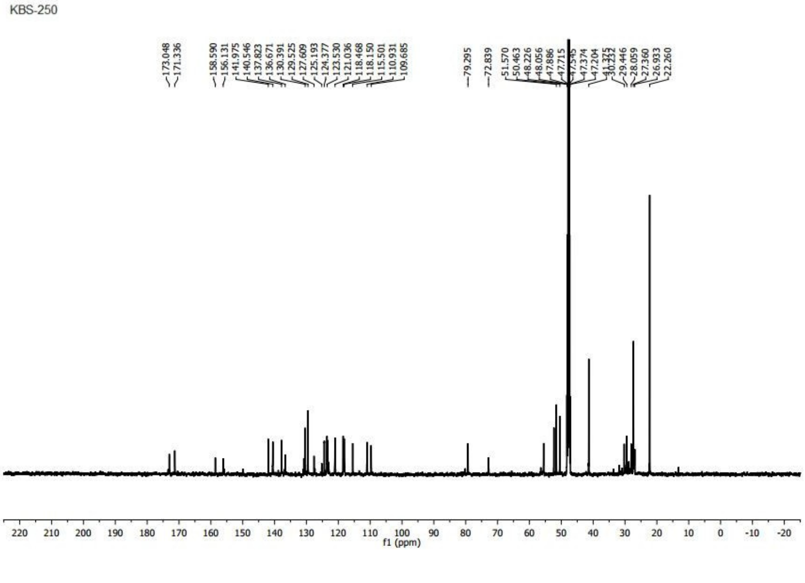

Figure S27. Boc-Trp-His[1-(3-trifluoromethylbenzyl)-5-iodo]-OMe (**9e**).

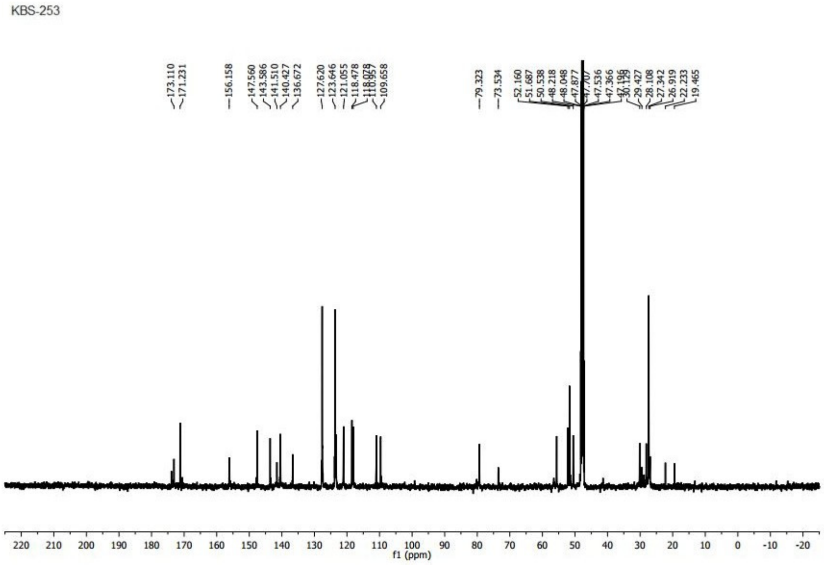

Figure S28. Boc-Trp-His[1-(4-nitrobenzyl)-5-iodo]-OMe (**9h**).

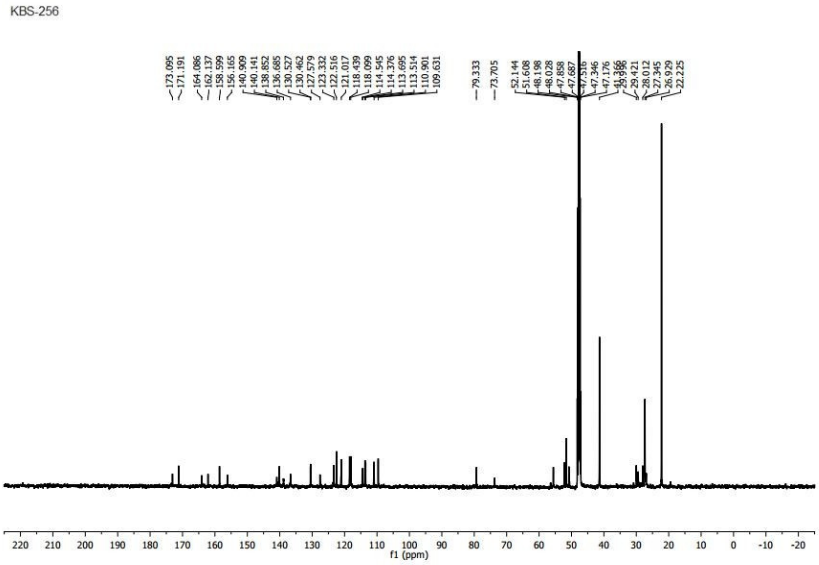

Figure S29. Boc-Trp-His[1-(3-fluorobenzyl)-5-iodo]-OMe (**9k**).

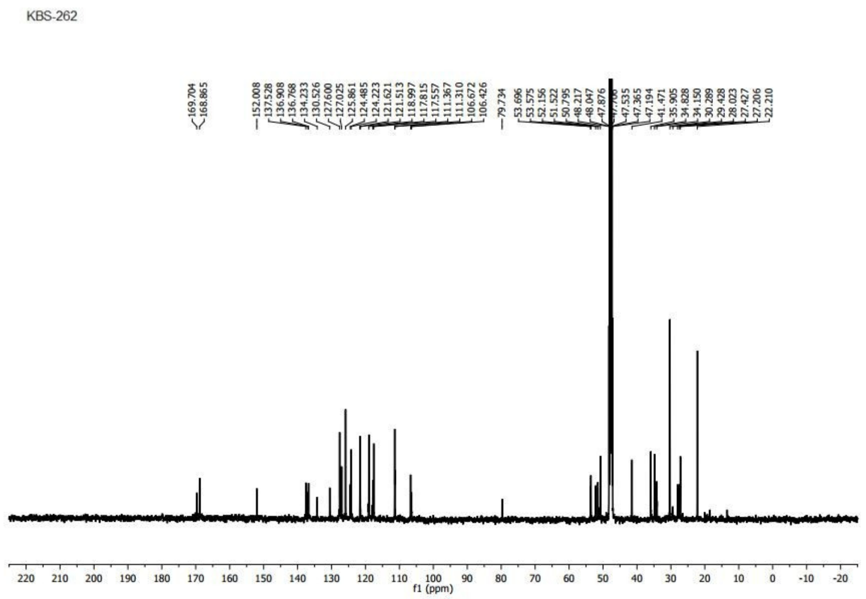

Figure S30. Trp-His[1-(4-*tert*-butylbenzyl)-5-iodo]-OMe (**10b**).

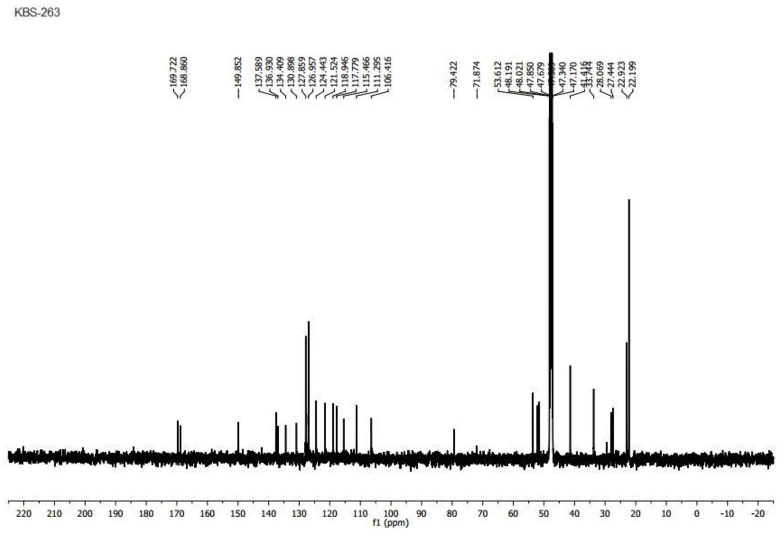

Figure S31. Trp-His[1-(4-*iso*-propylbenzyl)-5-iodo]-OMe (**10c**).

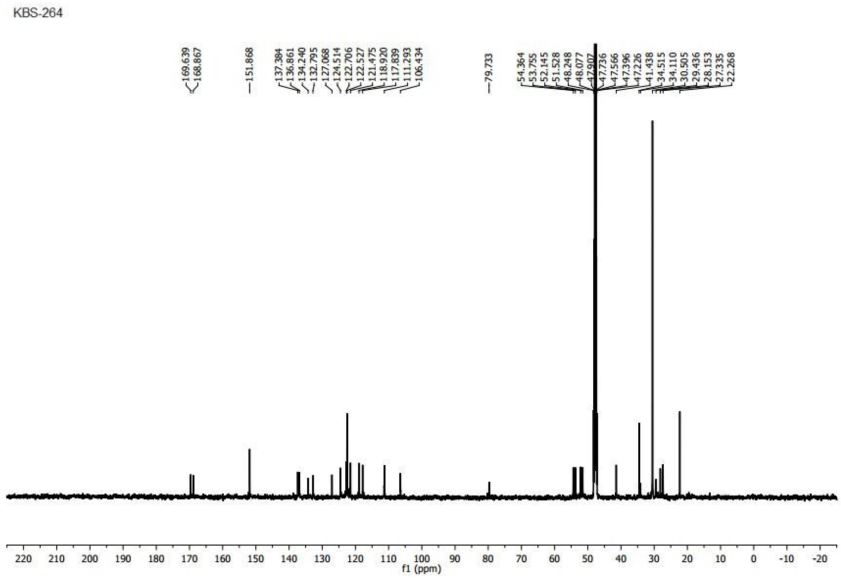

Figure S32. Trp-His[1-(3,5-di-*tert*-butylbenzyl)-5-iodo]-OMe (**10d**).

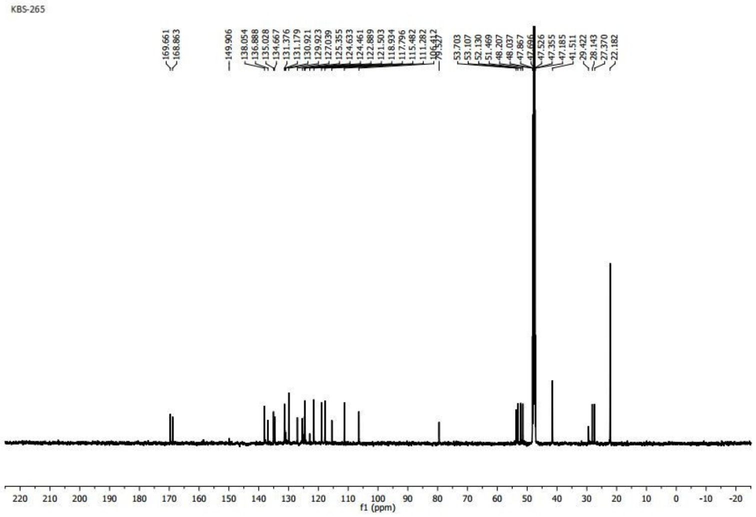

Figure S33. Trp-His[1-(3-trifluoromethylbenzyl)-5-iodo]-OMe (**10e**).

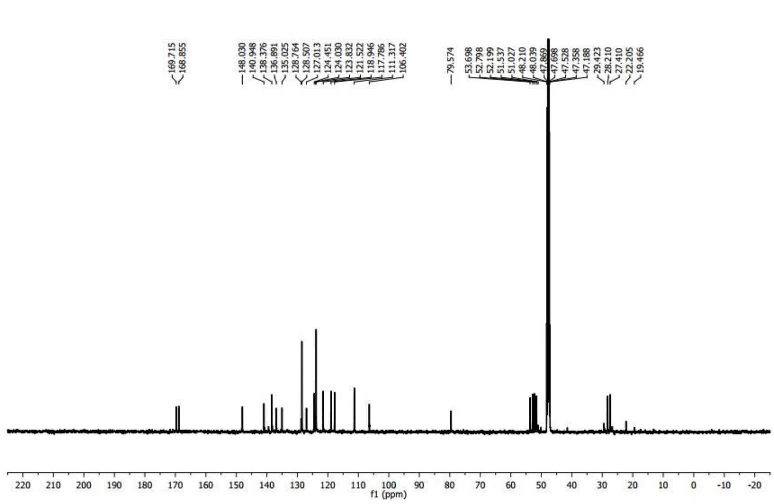

Figure S34. Trp-His[1-(4-nitrobenzyl)-5-iodo]-OMe (**10h**).

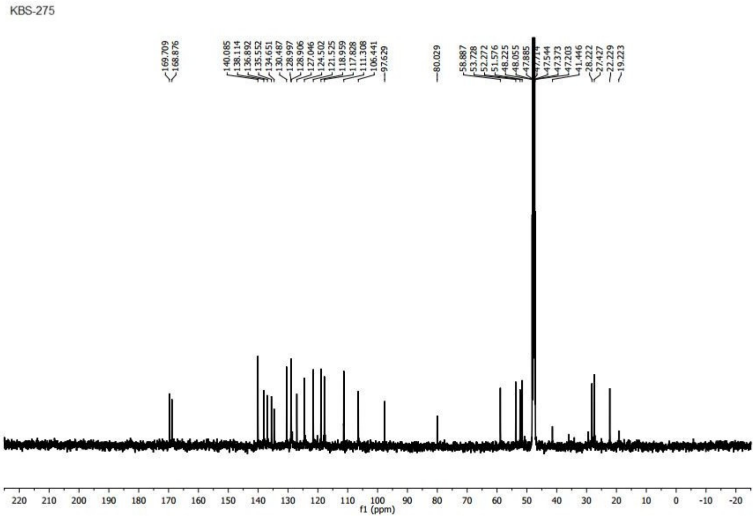

Figure S35. Trp-His[1-(2-iodobenzyl)-5-iodo]-OMe (**10o**).

4. HRMS Data of Representative Compounds

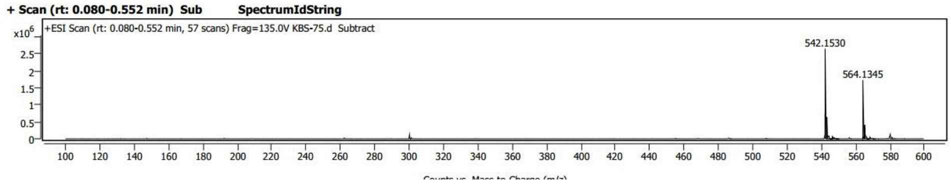

Figure S36. *N*-α-Boc-1-(4-*tert*-butylbenzyl)-5-iodo-L-histidine methyl ester (**7b**).

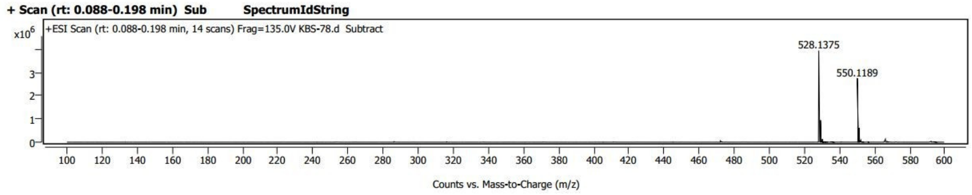

Figure S37. *N*-α-Boc-1-(4-*iso*-propylbenzyl)-5-iodo-L-histidine methyl ester (**7c**).

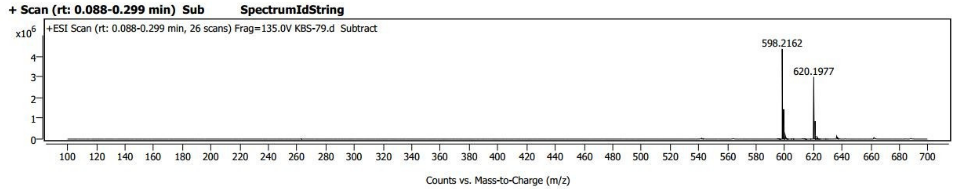

Figure S38. *N*-α-Boc-1-(3,5-di-*tert*-butylbenzyl)-5-iodo-L-histidine methyl ester (**7d**).

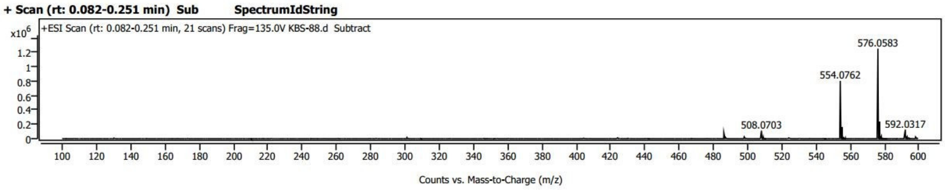

Figure S39. *N*-α-Boc-1-(3-trifluoromethylbenzyl)-5-iodo-L-histidine methyl ester (**7e**).

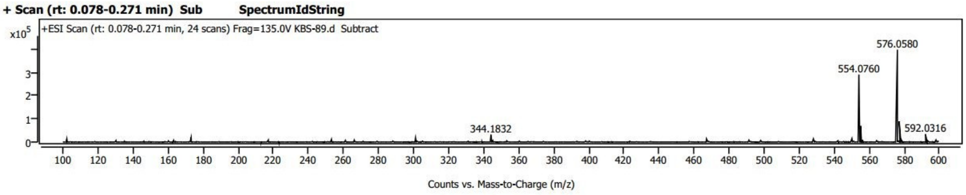

Figure S40. *N*-α-Boc-1-(2-trifluoromethylbenzyl)-5-iodo-L-histidine methyl ester (**7f**).

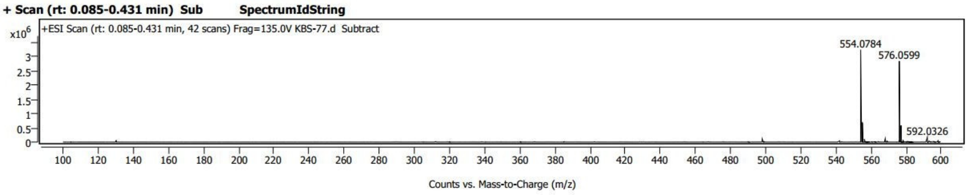

Figure S41. *N*-α-Boc-1-(4-trifluoromethylbenzyl)-5-iodo-L-histidine methyl ester (**7g**).

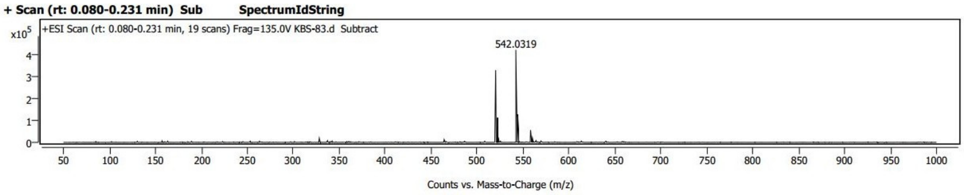

Figure S42. *N*-α-Boc-1-(3-chlorobenzyl)-5-iodo-L-histidine methyl ester (**7l**).

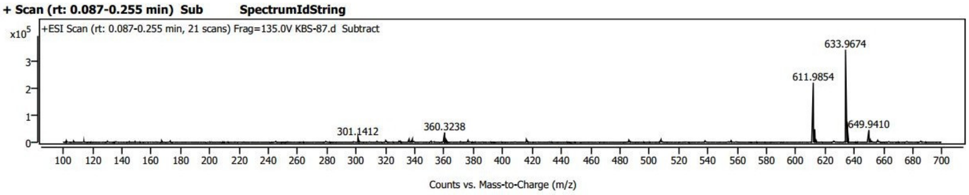

Figure S43. *N*-α-Boc-1-(3-iodobenzyl)-5-iodo-L-histidine methyl ester (**7n**).

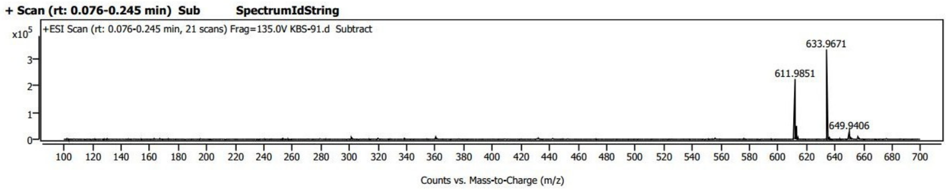

Figure S44. *N*-α-Boc-1-(2-iodobenzyl)-5-iodo-L-histidine methyl ester (**7o**).

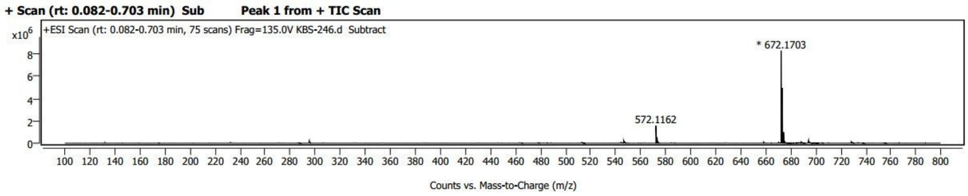

Figure S45. Boc-Trp-His(1-benzyl-5-iodo)-OMe (**9a**).

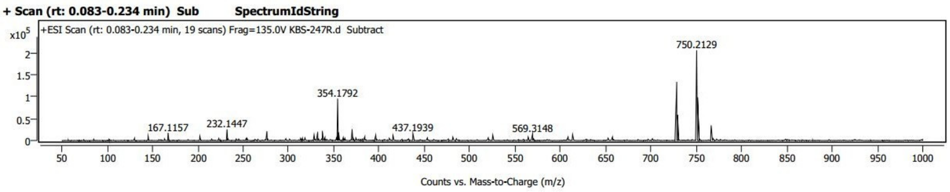

Figure S46. Boc-Trp-His[1-(4-*tert*-butylbenzyl)-5-iodo]-OMe (**9b**).

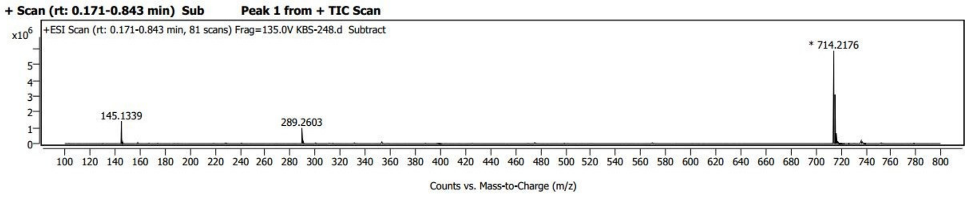

Figure S47. Boc-Trp-His[1-(4-*iso*-propylbenzyl)-5-iodo]-OMe (**9c**).

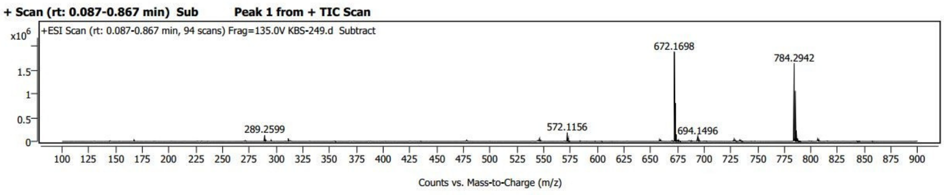

Figure S48. Boc-Trp-His[1-(3,5-di-*tert*-butylbenzyl)-5-iodo]-OMe (**9d**).

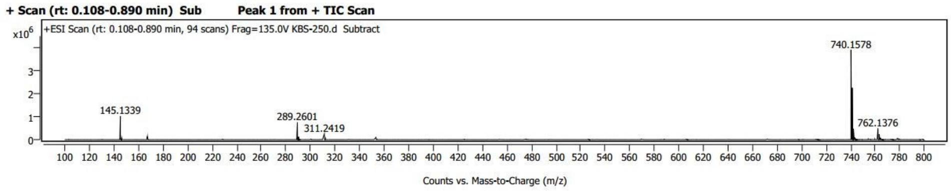

Figure S49. Boc-Trp-His[1-(3-trifluoromethylbenzyl)-5-iodo]-OMe (**9e**).

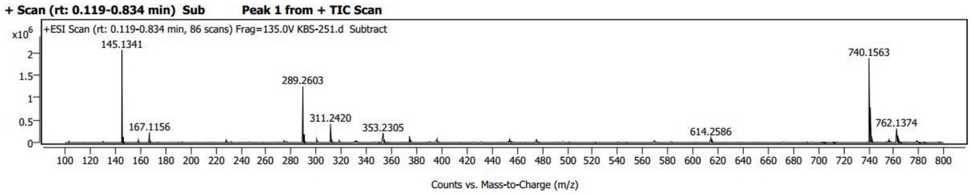

Figure S50. Boc-Trp-His[1-(2-trifluoromethylbenzyl)-5-iodo]-OMe (**9f**).

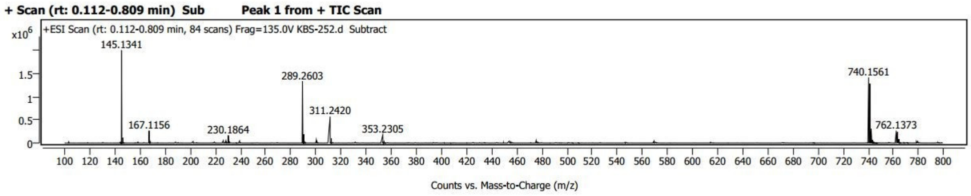

Figure S51. Boc-Trp-His[1-(4-trifluoromethylbenzyl)-5-iodo]-OMe (**9g**).

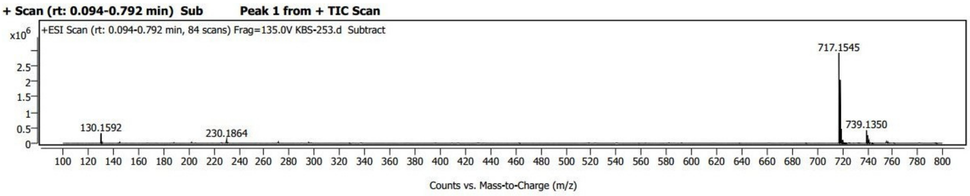

Figure S52. Boc-Trp-His[1-(4-nitrobenzyl)-5-iodo]-OMe (**9h**).

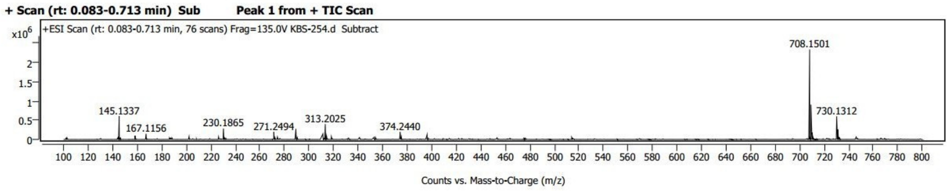

Figure S53. Boc-Trp-His[1-(3,4-difluorobenzyl)-5-iodo]-OMe (**9i**).

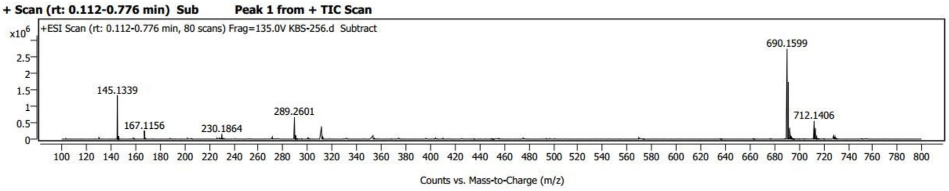

Figure S54. Boc-Trp-His[1-(3-fluorobenzyl)-5-iodo]-OMe (**9k**).

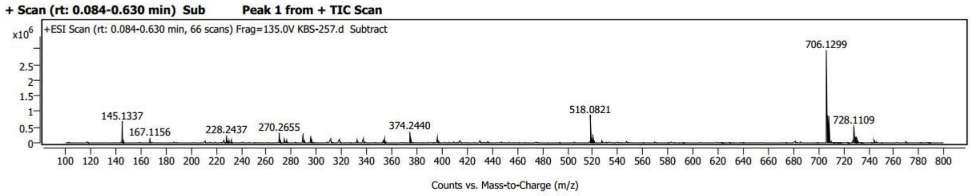

Figure S55. Boc-Trp-His[1-(3-chlorobenzyl)-5-iodo]-OMe (**9l**).

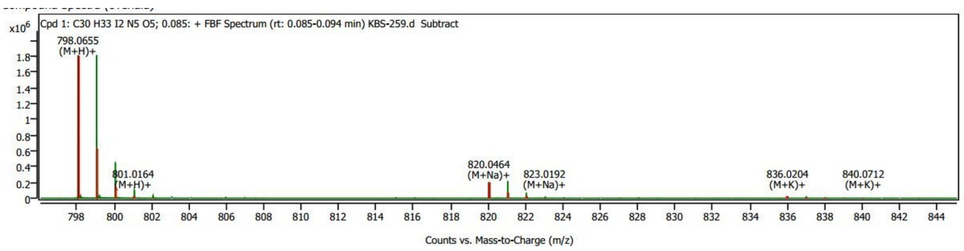

Figure S56. Boc-Trp-His[1-(3-iodobenzyl)-5-iodo]-OMe (**9n**).

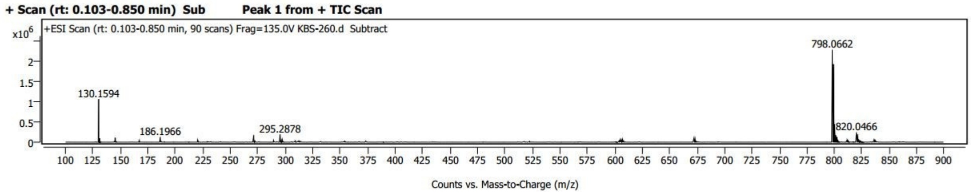

Figure S57. Boc-Trp-His[1-(2-iodobenzyl)-5-iodo]-OMe (**9o**).

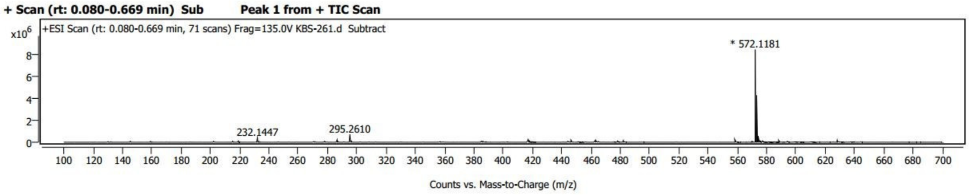

Figure S58. Trp-His(1-benzyl-5-iodo)-OMe (**10a**).

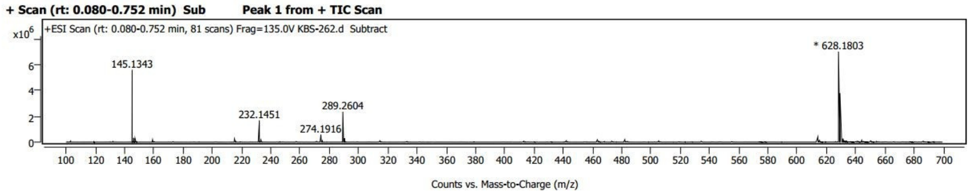

Figure S59. Trp-His[1-(4-*tert*-butylbenzyl)-5-iodo]-OMe (**10b**).

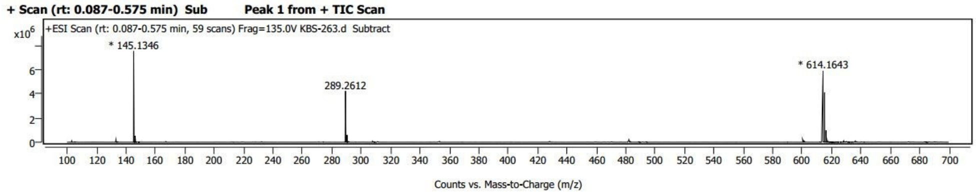

Figure S60. Trp-His[1-(4-*iso*-propylbenzyl)-5-iodo]-OMe (**10c**).

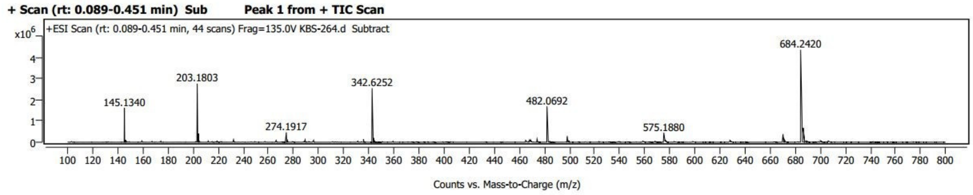

Figure S61. Trp-His[1-(3,5-di-*tert*-butylbenzyl)-5-iodo]-OMe (**10d**).

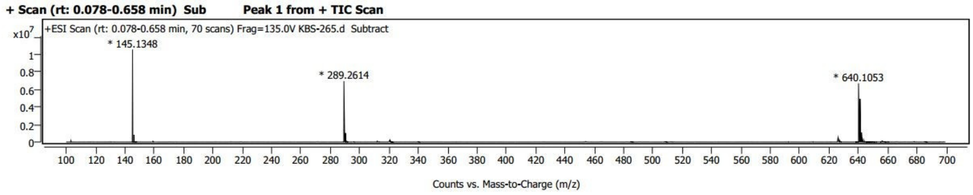

Figure S62. Trp-His[1-(3-trifluoromethylbenzyl)-5-iodo]-OMe (**10e**).

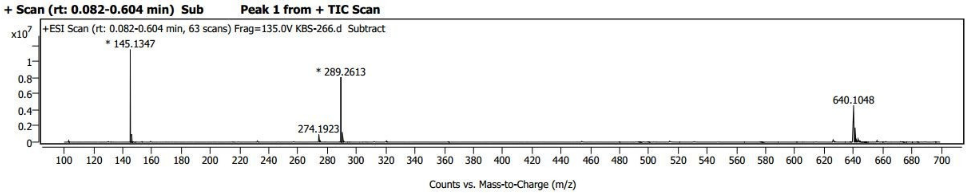

Figure S63. Trp-His[1-(2-trifluoromethylbenzyl)-5-iodo]-OMe (**10f**).

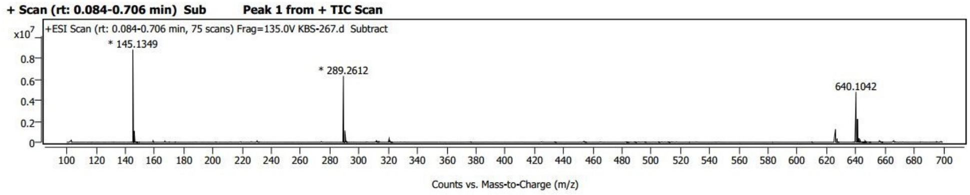

Figure S64. Trp-His[1-(4-trifluoromethylbenzyl)-5-iodo]-OMe (**10g**).

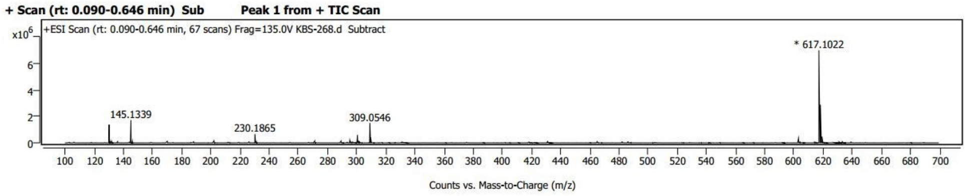

Figure S65. Trp-His[1-(4-nitrobenzyl)-5-iodo]-OMe (**10h**).

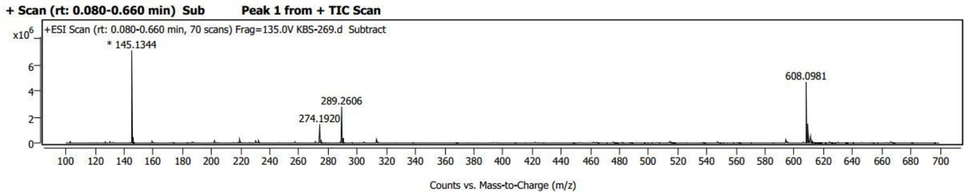

Figure S66. Trp-His[1-(3,4-difluorobenzyl)-5-iodo]-OMe (**10i**).

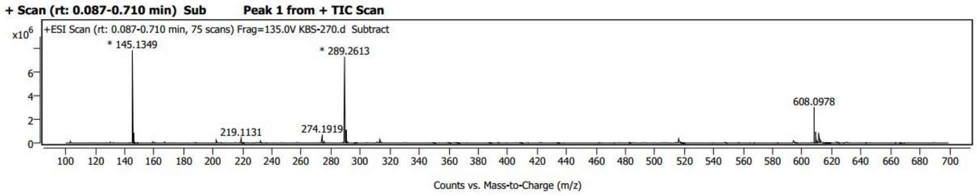

Figure S67. Trp-His[1-(3,5-difluorobenzyl)-5-iodo]-OMe (**10j**).

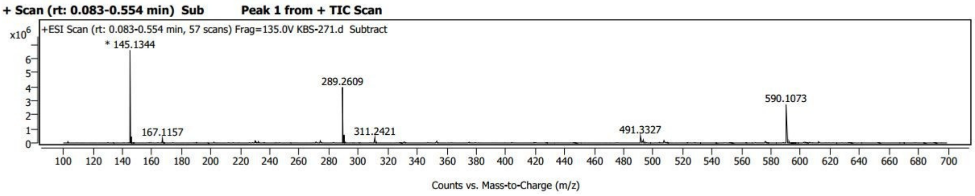

Figure S68. Trp-His[1-(3-fluorobenzyl)-5-iodo]-OMe (**10k**).

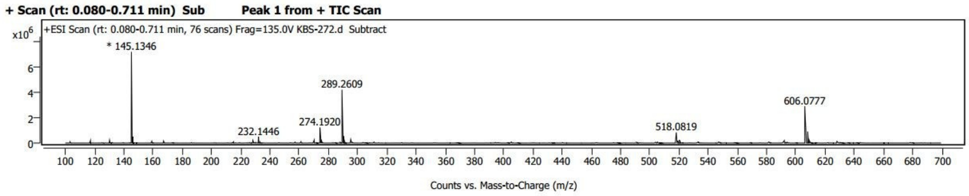

Figure S69. Trp-His[1-(3-chlorobenzyl)-5-iodo]-OMe (**10l**).

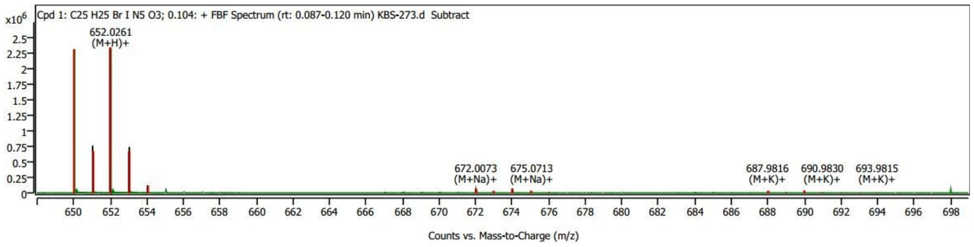

Figure S70. Trp-His[1-(3-bromobenzyl)-5-iodo]-OMe (**10m**).

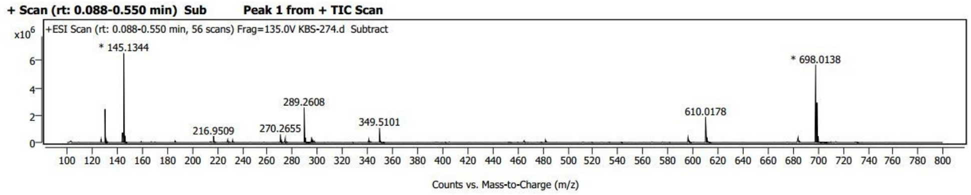

Figure S71. Trp-His[1-(3-iodobenzyl)-5-iodo]-OMe (**10n**).

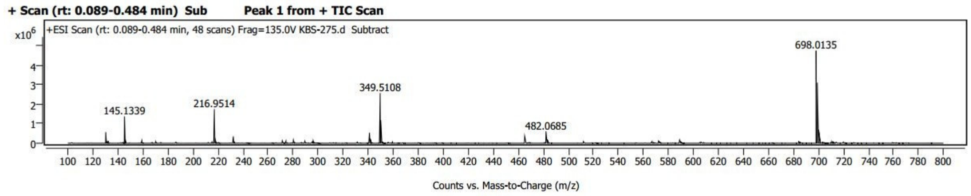

Figure S72. Trp-His[1-(2-iodobenzyl)-5-iodo]-OMe (**10o**).

5. HPLC Data of Representative Compounds

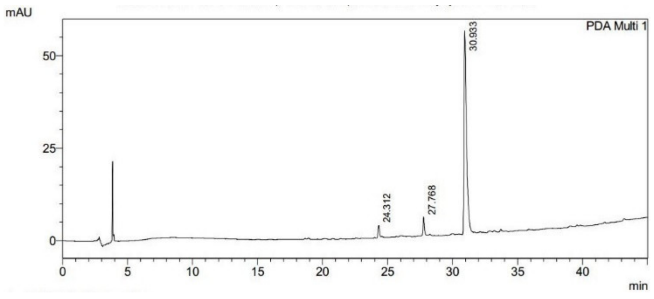

PeakTable

PDA Ch1 254nm 4nm

Peak#	Ret. Time	Area	Height	Area %
1	24.312	17466	2772	2.222
2	27.768	33243	4869	4.229
3	30.933	735387	54743	93.549
Total		786096	62383	100.000

Figure S73. Boc-Trp-His[1-(3,5-di-*tert*-butylbenzyl)-5-iodo]-OMe (**9d**).

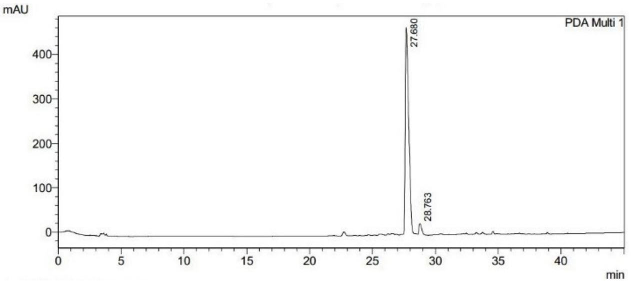

PeakTable

PDA Ch1 254nm 4nm

Peak#	Ret. Time	Area	Height	Height %	Area %
1	27.680	8917479	463584	95.561	96.923
2	28.763	283140	21534	4.439	3.077
Total		9200620	485118	100.000	100.000

Figure S74. Boc-Trp-His[1-(3-trifluoromethylbenzyl)-5-iodo]-OMe (**9e**).

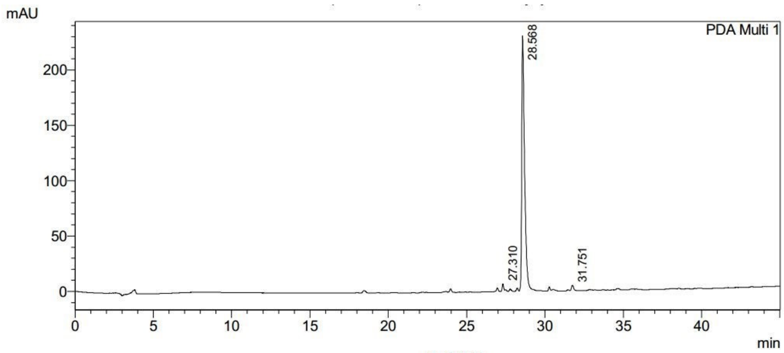

PeakTable

PDA Ch1 254nm 4nm

Peak#	Ret. Time	Area	Height	Area %
1	27.310	39097	6263	1.329
2	28.568	2873370	229234	97.676
3	31.751	29268	3883	0.995
Total		2941735	239380	100.000

Figure S75. Boc-Trp-His[1-(2-trifluoromethylbenzyl)-5-iodo]-OMe (**9f**).

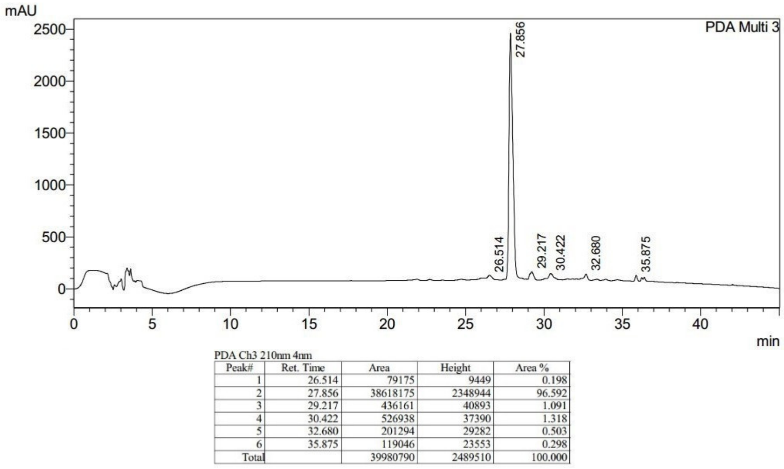

PDA Ch3 210nm 4nm

Peak#	Ret. Time	Area	Height	Area %
1	26.514	79175	9449	0.198
2	27.856	38618175	2348944	96.592
3	29.217	436161	40893	1.091
4	30.422	526938	37390	1.318
5	32.680	201294	29282	0.503
6	35.875	119046	23553	0.298
Total		39980790	2489510	100.000

Figure S76. Boc-Trp-His[1-(4-trifluoromethylbenzyl)-5-iodo]-OMe (**9g**).

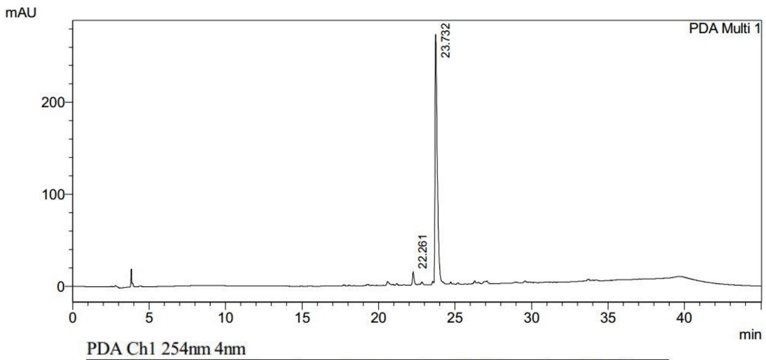

PDA Ch1 254nm 4nm

Peak#	Ret. Time	Area	Height	Area %
1	22.261	89029	13381	3.058
2	23.732	2822375	271422	96.942
Total		2911404	284803	100.000

Figure S77. Trp-His[1-(4-*iso*-propylbenzyl)-5-iodo]-OMe (**10c**).

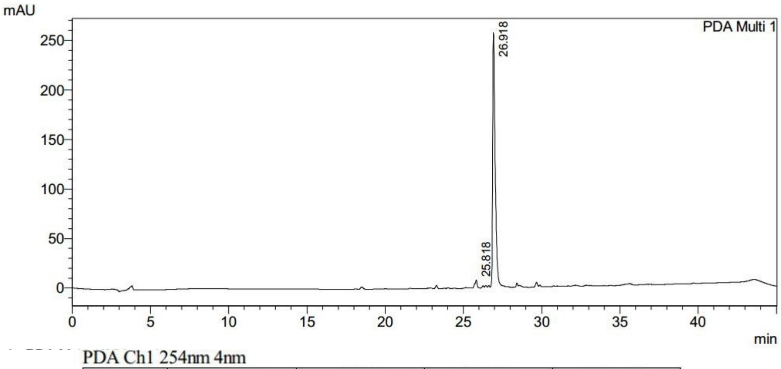

PDA Ch1 254nm 4nm

Peak#	Ret. Time	Area	Height	Area %
1	25.818	68125	7774	2.361
2	26.918	2817214	256056	97.639
Total		2885340	263830	100.000

Figure S78. Trp-His[1-(3,5-di-*tert*-butylbenzyl)-5-iodo]-OMe (**10d**).

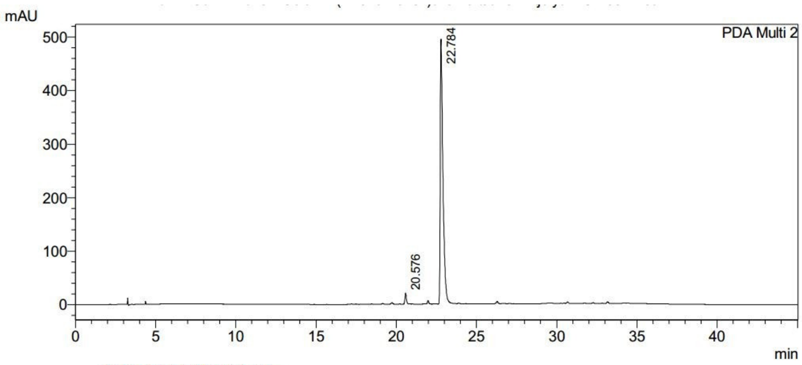

PDA Ch2 280nm 4nm

Peak#	Ret. Time	Area	Height	Area %
1	20.576	122932	20356	2.108
2	22.784	5709709	494213	97.892
Total		5832641	514568	100.000

Figure S79. Trp-His[1-(3,4-difluorobenzyl)-5-iodo]-OMe (**10i**).

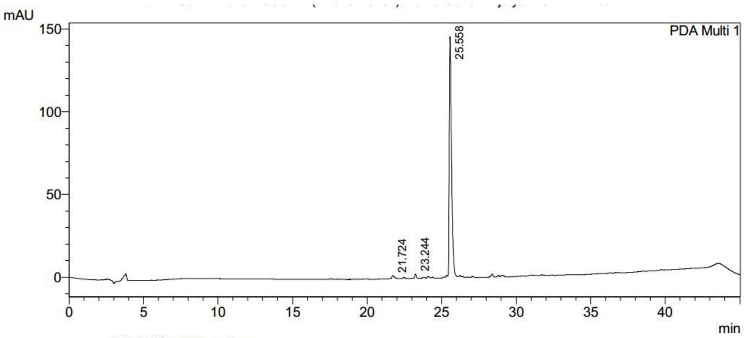

PDA Ch1 254nm 4nm

Peak#	Ret. Time	Area	Height	Area %
1	21.724	13498	1524	0.897
2	23.244	15969	2347	1.061
3	25.558	1475346	144417	98.042
Total		1504813	148289	100.000

Figure S80. Trp-His[1-(3-iodobenzyl)-5-iodo]-OMe (**10n**).

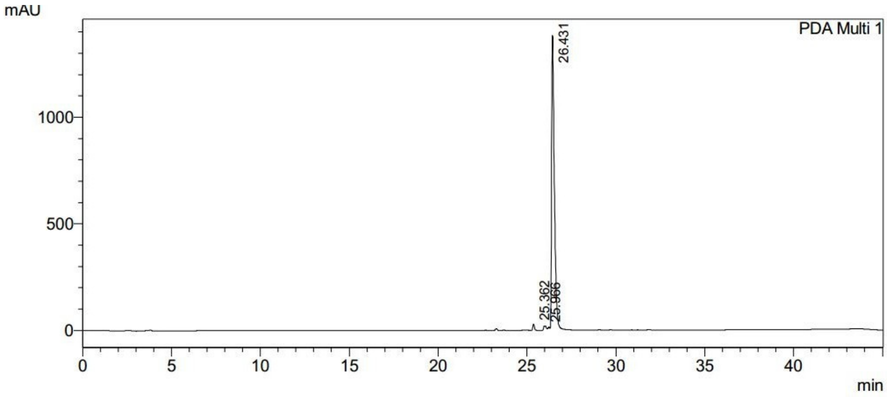

PDA Ch1 254nm 4nm

Peak#	Ret. Time	Area	Height	Area %
1	25.362	142516	27145	0.971
2	25.966	158291	18731	1.078
3	26.431	14379960	1372800	97.951
Total		14680767	1418676	100.000

Figure S81. Trp-His[1-(2-iodobenzyl)-5-iodo]-OMe (**10o**).

References

1. Abdo, M.-R.; Joseph, P.; Boigegrain, R.-A.; Liautard, J.-P.; Montero, J.-L.; Köhler, S.; Winum, J.-Y. Brucella suis histidinol dehydrogenase: Synthesis and inhibition studies of a series of substituted benzylic ketones derived from histidine. *Bioorganic Med. Chem*. **2007**, *15*, 4427–4433, https://doi.org/10.1016/j.bmc.2007.04.027.
2. Sharma, K.K.; Mandloi, M.; Jain, R. Regioselective copper-catalyzed N(1)-(hetero)arylation of protected histidine. *Org. Biomol. Chem.* **2016**, *14*, 8937–8941, https://doi.org/10.1039/c6ob01753c.
3. Mahindra, A.; Bagra, N.; Jain, R. Palladium-catalyzed regioselective C-5 arylation of protected L-histidine: microwave-assisted C–H activation adjacent to donor arm. *J. Org. Chem.* **2013**, *78*, 10954–10959.
4. Jain, R.; Avramovitch, B.; Cohen, L.A. Synthesis of ring-halogenated histidines and histamines. *Tetrahedron* **1998**, *54*, 3235–3242, https://doi.org/10.1016/s0040-4020(98)00068-4.
5. Meena, C.L.; Thakur, A.; Nandekar, P.P.; Sangamwar, A.T.; Sharma, S.S.; Jain, R. Synthesis of CNS active thyrotropin-releasing hormone (TRH)-like peptides: Biological evaluation and effect on cognitive impairment induced by cerebral ischemia in mice. *Bioorganic Med. Chem.* **2015**, *23*, 5641–5653, https://doi.org/10.1016/j.bmc.2015.07.022.
6. Mahindra, A.; Bagra, N.; Wangoo, N.; Jain, R.; Khan, S. I.; Jacob, M. R.; Jain, R. Syn-thetically modified L-histidine-rich peptidomimetics exhibit potent activity against Cryptococcus neoformans. *Bioorg. Med. Chem. Lett*. **2014**, *24*, 3150–3154.
